# Flower‐Like Amorphous MoO_3−_ *_x_* Stabilized Ru Single Atoms for Efficient Overall Water/Seawater Splitting

**DOI:** 10.1002/advs.202300342

**Published:** 2023-04-24

**Authors:** Dong Feng, Pengyan Wang, Rui Qin, Wenjie Shi, Lei Gong, Jiawei Zhu, Qianli Ma, Lei Chen, Jun Yu, Suli Liu, Shichun Mu

**Affiliations:** ^1^ State Key Laboratory of Advanced Technology for Materials Synthesis and Processing Wuhan University of Technology Wuhan 430070 China; ^2^ Foshan Xianhu Laboratory of the Advanced Energy Science and Technology Guangdong Laboratory Xianhu Hydrogen Valley Foshan 528200 China; ^3^ Key Laboratory of Advanced Functional Materials of Nanjing Nanjing Xiaozhuang University Nanjing 211171 China

**Keywords:** amorphous substrate, bifunctional catalysis, Ru single atom, water splitting

## Abstract

Benefitting from the maximum atom utilization efficiency, special size quantum effects and tailored active sites, single‐atom catalysts (SACs) have been promising candidates for bifunctional catalysts toward water splitting. Besides, due to the unique structure and properties, some amorphous materials have been found to possess better performance than their crystalline counterparts in electrocatalytic water splitting. Herein, by combining the advantages of ruthenium (Ru) single atoms and amorphous substrates, amorphous molybdenum‐based oxide stabilized single‐atomic‐site Ru (Ru SAs‐MoO_3−_ *_x_*/NF) catalysts are conceived as a self‐supported electrode. By virtue of the large surface area, enhanced intrinsic activity and fast reaction kinetics, the as‐prepared Ru SAs‐MoO_3−_ *_x_*/NF electrode effectively drives both oxygen evolution reaction (209 mV @ 10 mA cm^−2^) and hydrogen evolution reaction (36 mV @ 10 mA cm^−2^) in alkaline media. Impressively, the assembled electrolyzer merely requires an ultralow cell voltage of 1.487 V to deliver the current density of 10 mA cm^−2^. Furthermore, such an electrode also exhibits a great application potential in alkaline seawater electrolysis, achieving a current density of 100 mA cm^−2^ at a low cell voltage of 1.759 V. In addition, Ru SAs‐MoO_3−_ *_x_*/NF only has very small current density decay in the long‐term constant current water splitting test.

## Graphical abstract

A highly active bifunctional catalyst flower‐like Ru SAs‐MoO_3−_ *_x_*/NF is designed and constructed. Benefitting from the combination of ruthenium (Ru) single atoms and amorphous substrates, the as‐prepared Ru SAs‐MoO_3−_ *_x_*/NF electrode possesses excellent oxygen evolution reaction/hydrogen evolution reaction bifunctional activity and robust stability in both alkaline and alkaline seawater media, demonstrating excellent application prospect in practical multiscenario water electrolysis apparatuses.

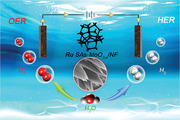

## 1 Introduction

The ever‐growing consumption of traditional fossil fuels and the environmental issues caused have promoted the development of renewable energy.^[^ ^1^, ^2^ ^]^ Among them, due to the high energy density and zero emission, hydrogen has arisen as a favorable alternative to fossil fuels.^[^ ^3^ ^]^ Electrochemical water electrolysis, utilizing the freshwater or more challenging low‐grade and saline surface water (such as seawater media) as feedstock, offers an economic and efficient strategy to produce high‐quality green hydrogen without carbon emission.^[^ ^4^, ^5^, ^6^ ^]^ However, the sluggish kinetics of the oxygen evolution reaction (OER) and hydrogen evolution reaction (HER) leads to extremely high electrode polarization, hence requiring high‐efficiency catalysts to reduce the dynamic overpotentials.^[^ ^7^, ^8^ ^]^ Owing to the outstanding catalytic activity, Pt‐based materials and Ir/Ru oxides have become the benchmark catalysts for HER and OER, respectively. However, their large‐scale application is severely restricted by expensive cost and scarce resource.^[^ ^9^, ^10^ ^]^ Therefore, it is urgent to lower the dosage of Pt group metals by a series of strategies such as construction of single‐atom catalysts (SACs).^[^ ^11^ ^]^ Due to the theoretically high efficiency and adjustable chemical reactivity, Ni‐based catalysts have become state‐of‐the‐art OER electrocatalysts in alkaline solutions.^[^ ^12^ ^]^ Nevertheless, in view of its poor HER performance, the Mo‐based catalyst with variable valence states becomes the better choice for the overall water/seawater splitting as bifunctional catalyst.^[^ ^13^ ^]^

As a cost‐effective precious metal, ruthenium (Ru) possesses Pt‐like hydrogen binding energy and superior ability for adsorption of OH^—^ as well as the dissociation of water.^[^ ^14^, ^15^ ^]^ Downsizing the Ru nanoparticles (NPs) into nanoclusters and even single atoms can significantly increase the catalytic activity and selectivity toward electrochemical reactions.^[^ ^16^, ^17^ ^]^ Due to the maximum atom utilization efficiency, special size quantum effects and tailored active sites, SACs have been regarded as promising bifunctional catalysts for toward water splitting.^[^ ^18^, ^19^, ^20^ ^]^ However, since the coordination environment of stabilized single atoms is highly dependent on the local electronic structure of defect sites in substrates, the activity of SACs is also greatly affected by the substrate materials.^[^ ^21^, ^22^, ^23^ ^]^ Among various substrates, transition metal oxides (TMOs) have attracted intense attention because of good electronic conductivity, robust durability and excellent corrosion resistivity, thus high catalytic performance can be expected.^[^ ^24^, ^25^, ^26^ ^]^ Moreover, the abundant defect sites (such as steps, corners, vacancies) and —OH groups on substrate surfaces of TMOs can also serve as the anchoring site for single metal atoms.^[^ ^27^ ^]^

Furthermore, due to the disorder long‐distance arrangement and unsaturated coordination environment, the amorphous materials are attracting growing attentions in diverse fields.^[^ ^28^, ^29^ ^]^ More importantly, compared to their crystalline counterparts, they own a larger number of exposed surfaces and defects, which provides abundant active sites, facilitating the transportation of charged species during the electrochemical water splitting.^[^ ^30^, ^31^ ^]^ In addition, their flexibility allows them to self‐regulate according to the electrocatalytic conditions, enabling both surface and volume confined electrocatalysis.^[^ ^32^, ^33^ ^]^ Therefore, the construction of amorphous structures has been regarded as a promising strategy to boost the bifunctional activity of catalysts.

Based on the above inspiration, we design and build an amorphous molybdenum oxide fixed single‐atomic‐site Ru catalyst (Ru SAs‐MoO_3−_ *_x_*/NF). The 3D flower‐like morphology, amorphous feature, and uniform distribution of single Ru atoms endow Ru SAs‐MoO_3−_ *_x_*/NF with abundant active sites and excellent conductivity. As a result, the as‐prepared catalyst exhibits outstanding bifunctional electrocatalytic activity and durability toward both OER and HER in alkaline solutions. Furthermore, the assembled electrolyzer with the catalyst reveals high‐performance water/seawater splitting.

## 2 Results and Discussion

### 2.1 Synthesis and Structural Characterization

**Figure 1:**
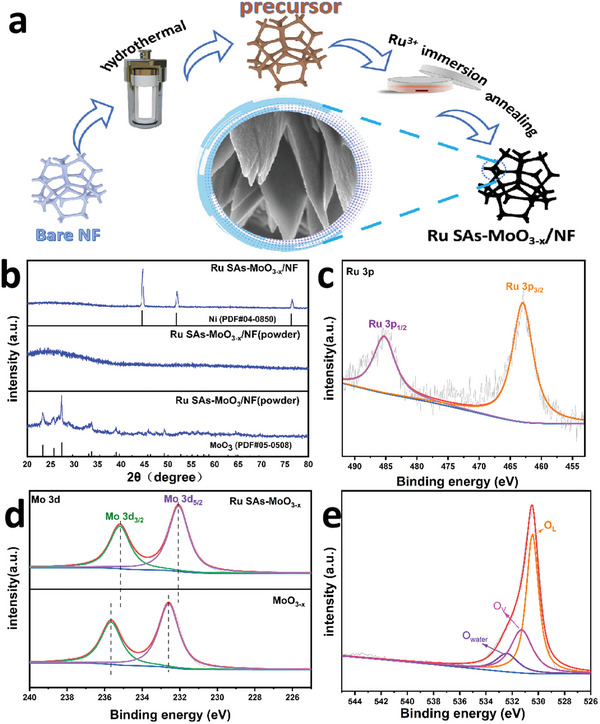
a) Schematic illustration for the fabrication of the Ru SAs‐MoO_3−_ *_x_*/NF. b) XRD patterns of Ru SAs‐MoO_3−_ *_x_*/NF and Ru SAs‐MoO_3_/NF. c) Ru 3p and e) O 1s deconvoluted spectra of Ru SAs‐MoO_3−_ *_x_*/NF. d) High‐resolution XPS spectra for Mo 3d of Ru SAs‐MoO_3−_ *_x_*/NF and MoO_3−_ *_x_*/NF.

As illustrated in **Figure** **1**a, a three‐step procedure was utilized to construct Ru SAs‐MoO_3−_ *_x_* nanoflakes. Specifically, the precursor was first grown on the surface of nickel foam (NF) through a hydrothermal process, and then the as‐prepared precursor was immersed in RuCl_3_ solutions to introduce Ru atoms. Finally, the product was obtained by annealed at 350 °C for 2 h in air atmosphere. For comparison, the crystalline counterpart Ru SAs‐MoO_3_/NF was synthesized through a similar pathway except for changing the annealing temperature to 450 °C.

As presented in Figure 1b, for Ru SAs‐MoO_3−_ *_x_*/NF only three X‐ray diffraction (XRD) characteristic peaks corresponding to the NF substrate (PDF#04‐0850) can be detected. Moreover, no obvious characteristic peaks are exhibited for the powder scraped from Ru SAs‐MoO_3−_ *_x_* NF, illustrating that the substrate of Ru SAs exists in the form of amorphous state. With the increase of annealing temperature, the crystalline MoO_3_ occurs at about 400 °C (Figure S1a, Supporting Information). By contrast with the XRD patterns in Figure S1b (Supporting Information), it indicates that the incorporation of fractional Ru cannot affect the crystallization process. The XRD pattern of the powder scraped from Ru SAs‐MoO_3_/NF can be well assigned to the crystalline MoO_3_ (PDF#05‐0508), demonstrating its crystalline structure.

From X‐ray photoelectron spectroscopy (XPS) spectra (Figure S2, Supporting Information), it shows the coexistence of Ru, Mo, O, C, and Ni elements. Among them, the C and Ni signals are originated from the surface adsorption and NF as substrate, respectively (Figure S3, Supporting Information). As for the deconvoluted spectrum of Ru 3p state for Ru SAs‐MoO_3−_ *_x_*/NF (Figure 1c), the doublet peaks at 463.11 and 485.26 eV can be attributed to the Ru 3p_3/2_ and Ru 3p_1/2_, respectively, suggesting the existence of Ru in the sample with a valence state of +*δ* (0 < *δ* < 4).^[^ ^34^, ^35^ ^]^ Importantly, the proportion of Ru was determined to be 2.50 wt% (average value, ±0.11 wt%) measured by the inductively coupled plasma (ICP). Such a low loading of Ru undoubtedly can reduce the cost of catalysts. The similar oxidation state and content (2.40 wt%) of Ru in the crystalline counterpart Ru SAs‐MoO_3_/NF reveals that different annealing conditions cannot change the existing form of Ru (Figure S4a, Supporting Information). As displayed in Figure 1d, the Mo 3d spectrum of Ru SAs‐MoO_3−_ *_x_*/NF reveals two strong peaks centered at 232.07 and 235.19 eV, which can be assigned to Mo 3d_5/2_ and Mo 3d_3/2_, respectively.^[^ ^36^, ^37^ ^]^ Compared to the crystalline counterpart Ru SAs‐MoO_3_/NF, the valence of Mo in Ru SAs‐MoO_3−_ *_x_*/NF is lower, suggesting the formation of oxygen vacancies (Figure S4b, Supporting Information).^[^ ^38^ ^]^ Notably, there is a negative shift of Mo 3d signals compared to that of pure MoO_3−_ *_x_*/NF, indicating that the introduction of Ru led to the increased electron cloud density as well as the enhanced electronegative charge.^[^ ^39^, ^40^ ^]^ As shown in Figure 1e, the O 1s spectrum of Ru SAs‐MoO_3−_ *_x_*/NF can be deconvoluted into three subpeaks at 530.45, 531.28, and 532.40 eV, assigned to lattice O in oxides, oxygen vacancies, and absorption water, respectively.^[^ ^41^, ^42^ ^]^ Compared to the crystalline counterpart, the peak of oxygen vacancies in Ru SAs‐MoO_3−_ *_x_*/NF is higher, demonstrating the rich oxygen vacancy in the amorphous substrate (Figure S4c, Supporting Information).

**Figure 2:**
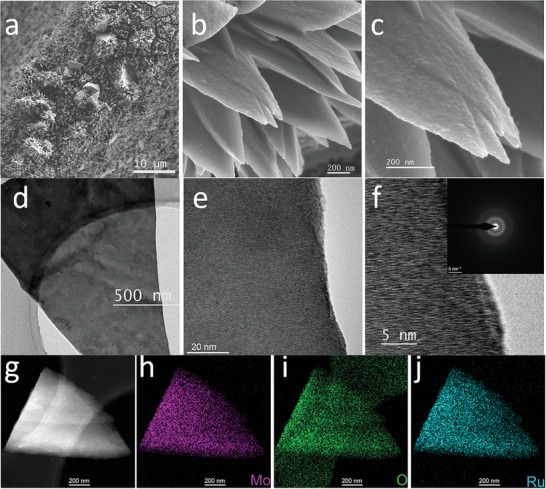
a–c) FESEM images of Ru SAs‐MoO_3−_ *_x_*/NF. d–f) Low‐ and high‐resolution TEM images and inserted FFT ring pattern of Ru SAs‐MoO_3−_ *_x_*/NF. g–j) HAADF image and corresponding EDX elemental mappings of Ru SAs‐MoO_3−_ *_x_*/NF.

**Figure 3:**
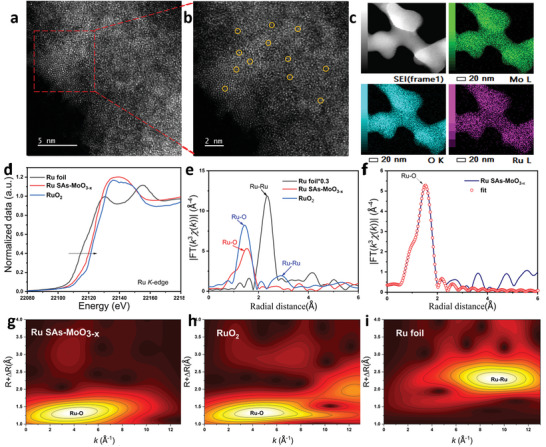
Structure characterization of Ru single atom in Ru SAs‐MoO_3−_ *_x_*. a,b) HAADF‐STEM images of Ru SAs‐MoO_3−_ *_x_*. c) HAADF‐STEM image and related elemental mapping images of Ru SAs‐MoO_3−_ *_x_*. d,e) Ru K‐edge XANES and EXAFS for Ru SAs‐MoO_3−_ *_x_*, RuO_2_, and Ru foil. f) R‐space fitting curve for Ru SAs‐MoO_3−_ *_x_*. g–i) WT for the k_2_‐weighted EXAFS signal for Ru SAs‐MoO_3−_ *_x_*, RuO_2_, and Ru foil.

Field emission scanning electron microscopy (FESEM) images (Figure S5, Supporting Information) show that, different from the smooth surface of bare NF, nanoflake arrays were uniformly grown on the surface of NF substrate during the hydrothermal process. Furthermore, it can be found that the Ru‐incorporation and annealing did not change the morphology of catalysts (Figure S6, Supporting Information). Specifically, Ru SAs‐MoO_3−_ *_x_*/NF exhibits a 3D flower‐like morphology composed of interconnected nanoflakes (**Figure** **2**a–c). Such an open structure endows the material with larger large surface area and more catalytic active sites, promoting the mass transfer kinetics for electrochemical water splitting.^[^ ^43^, ^44^ ^]^ As depicted in Figure 2d, the single flake of flower‐like structure is further presented in the transmission electron microscopy (TEM) image. The amorphous feature of Ru SAs‐MoO_3−_ *_x_* can be confirmed by the absence of lattice fringes in the HRTEM images (Figure 2e,f) and the rings in the fast Fourier transform (FFT) pattern (inset in Figure 2f). Combining the XRD analysis results, it can be concluded that there exists an amorphous MoO_3−_ *_x_* substrate. The uniform distribution of elemental Mo, O, and Ru inside a single flake is illustrated by the energy‐dispersive X‐ray spectroscopy (EDX) elemental mapping images in Figure 2g–j. As displayed in **Figure** **3**a–c, the high‐resolution images of high‐angle annular dark‐field (HAADF) shows that individual Ru atoms as distinguishable bright spots labeled by yellow circles are uniformly dispersed over the amorphous MoO_3−_ *_x_* substrate.

Furthermore, the X‐ray absorption spectroscopy (XAS) characterizations were conducted to determine the valance states and local coordination structures of Ru SAs‐MoO_3−_ *_x_*. As shown in Figure 3d, X‐ray absorption near‐edge spectra (XANES) at Ru K‐edge confirm that the edge energy (E0) for Ru SAs‐MoO_3−_ *_x_* is lower than that of RuO_2_, while higher than that of Ru foil, suggesting Ru atoms carry positive charges +*δ* (0 < *δ* < 4), consistent with the XPS characterization results.^[^ ^45^, ^46^ ^]^ The Fourier transformed (FT) k^3^‐weighted extended X‐ray absorption fine structure (EXAFS) spectrum of Ru SAs‐MoO_3−_ *_x_* shows a distinct peak at 2.02 Å, which should be assigned to the Ru—O bond in Ru SAs‐MoO_3−_ *_x_* (Figure 3e). Moreover, the fitting EXAFS result of R‐space spectrum for Ru SAs‐MoO_3−_ *_x_* indicates that each Ru atom is coordinated with five oxygen atoms (Figure 3f; and Table S1, Supporting Information). As displayed in Figure 3g–i, the wavelet transform (WT) of Ru K‐edge EXAFS exhibits a main peak at about 4.0 Å^−1^ that can be attributed to the Ru—O coordination for Ru SAs‐MoO_3−_ *_x_*. In addition, the Mo K‐edge XANES spectrum confirms that the Mo cation in Ru SAs‐MoO_3−_ *_x_* has a lower oxidation state than that in crystalline MoO_3_ (Figure S7, Supporting Information). The coordination environment of Mo atoms in Ru SAs‐MoO_3−_ *_x_* is revealed by the EXAFS results (Figure S8 and Table S1, Supporting Information), the total coordination number of Mo—O is obviously lower than 6 in standard MoO_3_ sample, illustrating the presence of a large amount of oxygen vacancy defects caused by the amorphous strategy and the Ru introduction. Notably, as shown in the WT spectra (Figure S9, Supporting Information), there is a slight signal of the Mo—Ru bond in Ru SAs‐MoO_3−_ *_x_* rather than the Mo—Mo bond in MoO_3_, which can be attributed to the occupation of some Ru atoms at Mo atomic sites.^[^ ^47^ ^]^ Based on all the above characterization results, the inference that Ru single atoms are stabilized by flower‐like amorphous MoO_3−_ *_x_* substrate is further confirmed.

### 2.2 Electrochemical Evaluation for OER

**Figure 4:**
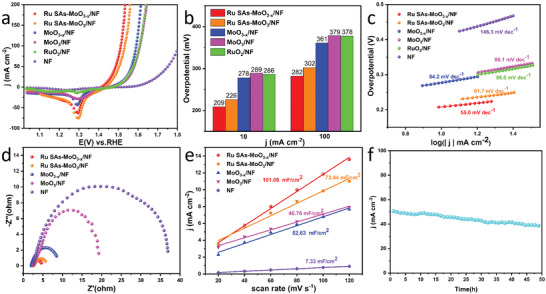
a) OER polarization curves with iR‐compensation for Ru SAs‐MoO_3−_ *_x_*/NF, Ru SAs‐MoO_3_/NF, MoO_3−_ *_x_*/NF, MoO_3_/NF, bare NF, and RuO_2_ on NF in 1 m KOH recorded at 5 mV s^−1^. b) Corresponding overpotential of 10 and 100 mA cm^−2^. c) Corresponding Tafel plots. d) EIS plots of Ru SAs‐MoO_3−_ *_x_*/NF, Ru SAs‐MoO_3_/NF, MoO_3−_ *_x_*/NF, MoO_3_/NF, and bare NF. e) Double‐layer capacitance (*C*_dl_) of Ru SAs‐MoO_3−_ *_x_*/NF, Ru SAs‐MoO_3_/NF, MoO_3−_ *_x_*/NF, MoO_3_/NF, and bare NF. f) Long‐term *i*–*t* response test for Ru SAs‐MoO_3−_ *_x_*/NF.

The OER performance of Ru SAs‐MoO_3−_ *_x_*/NF, Ru SAs‐MoO_3_/NF, MoO_3−_ *_x_*/NF, MoO_3_/NF, RuO_2_/NF, and bare NF was first investigated in alkaline media (1 m KOH, pH = 14) with a scan rate of 5 mV s^−1^. The linear sweep voltammetry (LSV) curves with iR‐compensated are displayed in **Figure** **4**a, indicating the activity trend of Ru SAs‐MoO_3−_ *_x_*/NF > Ru SAs‐MoO_3_/NF > MoO_3−_ *_x_*/NF > RuO_2_/NF > MoO_3_/NF > bare NF. It can be seen that the amorphous structure plays a pivotal role in improving the OER electrocatalytic activity, and the Ru single atoms further enhance the OER performance. As shown in Figure 4b, Ru SAs‐MoO_3−_ *_x_*/NF electrode exhibits the lowest overpotential of 209 mV at the current density of 10 mA cm^−2^, while to deliver the same current density Ru SAs‐MoO_3_/NF, MoO_3−_ *_x_*/NF, MoO_3_/NF, and RuO_2_/NF need 226, 278, 289, and 286 mV, respectively. Likewise, the OER performance of Ru SAs‐MoO_3−_ *_x_*/NF remains ahead of control samples even at larger current densities. Importantly, the OER performance of Ru SAs‐MoO_3−_ *_x_*/NF is quite competitive as compared to the lately reported relevant catalysts (Table S3, Supporting Information).

As depicted in Figure 4c, the Tafel slopes of Ru SAs‐MoO_3−_ *_x_*/NF, Ru SAs‐MoO_3_/NF, MoO_3−_ *_x_*/NF, MoO_3_/NF, RuO_2_/NF, and bare NF are 55.0, 61.7, 84.2, 86.1, 86.0, 146.3 mV dec^−1^, respectively, confirming the more favorable reaction kinetics for Ru SAs‐MoO_3−_ *_x_*/NF electrode. Moreover, as revealed in Figure 4d; and Figure S10 (Supporting Information), Ru SAs‐MoO_3−_ *_x_*/NF possesses the smallest charge transfer resistance (*R*_ct_) of 2.3 Ω, suggesting an expeditious charge transfer. To explore the intrinsic activity of catalysts, the double‐layer capacitance (*C*_dl_) as electrochemical surface area (ECSA) was calculated through cyclic voltammetry (CV) scans under various scan rates (Figure S11, Supporting Information).^[^ ^48^ ^]^ As shown in Figure 4e, Ru SAs‐MoO_3−_ *_x_*/NF electrode possesses the largest *C*_dl_ value of 101.06 mF cm^−2^ as compared to that of Ru SAs‐MoO_3_/NF (73.44 mF cm^−2^), MoO_3−_ *_x_*/NF (52.63 mF cm^−2^), MoO_3_/NF (46.76 mF cm^−2^), and bare NF (7.33 mF cm^−2^), indicating that Ru SAs‐MoO_3−_ *_x_*/NF has the largest electrochemical surface area which provides the most reaction active sites, in accordance with its excellent OER performance. Accordingly, the ECSA normalized polarization curves were exhibited in Figure S12a (Supporting Information), Ru SAs‐MoO_3−_ *_x_*/NF electrode still exhibits the smallest overpotential under the same current density, demonstrating the highest intrinsic catalytic activity of Ru SAs‐MoO_3−_ *_x_*/NF toward OER. In addition, the number of active sites (*n*) was measured by CV tests (Figure S13a–d, Supporting Information). Then, the turnover frequency (TOF) values of Ru SAs‐MoO_3−_ *_x_*/NF, Ru SAs‐MoO_3_/NF, MoO_3−_ *_x_*/NF, and MoO_3_/NF were calculated in terms of the *n* value and compared in Figure S13e (Supporting Information). Particularly, Ru SAs‐MoO_3−_ *_x_*/NF possesses the highest TOF value (0.27 s^−1^) at an OER overpotential of 300 mV, displaying the optimized intrinsic activity toward OER.

Long‐term stability for Ru SAs‐MoO_3−_ *_x_*/NF during OER process was then tested through 20 000 CV cycles (Figure S14, Supporting Information). It can be found that its polarization curve remains almost unchanged after 20 000 CV cycles. Moreover, as shown in Figure 4f, at a constant current density of 50 mA cm^−2^ during 50 h testing, the chronoamperometric curve of Ru SAs‐MoO_3−_ *_x_*/NF exhibits a negligible change, with activity loss of 0.25 mA cm^−2^ h^−1^, much lower than that of commercial RuO_2_ on NF (1.47 mA cm^−2^ h^−1^), as shown in Figure S15 (Supporting Information). All these results prove the excellent stability of Ru SAs‐MoO_3−_ *_x_*/NF. After the OER stability test, the composition, morphology and chemical states of Ru SAs‐MoO_3−_ *_x_*/NF electrode were analyzed to further gain insights into the reaction mechanism. There was still no obvious characteristic peak in the XRD pattern of the powder scraped from the working electrode after OER test (Figure S16, Supporting Information). As displayed in Figure S17a (Supporting Information), the FESEM image of Ru SAs‐MoO_3−_ *_x_*/NF retains the pristine 3D flower‐like morphology composed by nanoflakes with a rougher face. The XPS spectrum after durability test is revealed in Figure S17b–d (Supporting Information). The Mo 3d spectrum after OER testing remains nearly the same as the initial sample except for the slightly reduced intensity. Besides, the relatively increased content of O_water_ demonstrates the advantageous adsorption of intermediates on the amorphous surface during the OER process. Notably, a positive shift can be observed in the Ru 3p signal, suggesting the slight valence ascension caused by the oxidation of Ru single atoms.^[^ ^49^, ^50^ ^]^ Besides, the dissolutive content of metallic moieties in the electrolyte during durability test was evaluated by ICP, revealing about 20.6% dissolution of Ru (0.045 mg L^−1^). Therefore, the slight attenuation of OER performance for Ru SAs‐MoO_3−_ *_x_*/NF during the long‐term stability testing can be attributed to the partial dissolution of Ru species and the destruction of morphology.

### 2.3 Electrochemical Evaluation for HER

**Figure 5:**
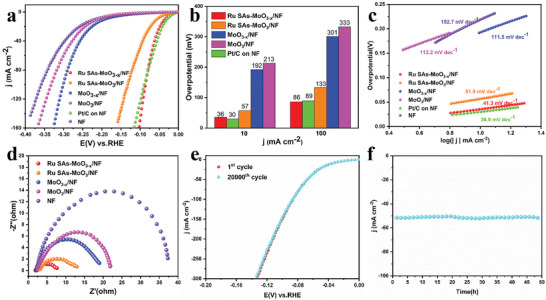
a) HER polarization curves with iR‐compensation for as‐prepared electrode materials and commercial Pt/C catalyst on NF in 1 m KOH recorded at 5 mV s^−1^. b) Corresponding overpotential at *j* = 10 and 100 mA cm^−2^. c) Corresponding Tafel plots. d) EIS plots of different electrodes. e) Polarization curves recorded before and after 20 000 CV cycles for Ru SAs‐MoO_3−_ *_x_*/NF. f) Long‐term *i*–*t* response test for Ru SAs‐MoO_3−_ *_x_*/NF.

Next, the electrocatalytic HER activity was examined in the same alkaline electrolyte. As depicted in **Figure** **5**a, iR‐compensated polarization curves were obtained to evaluate the HER performance of the catalysts. As shown in Figure 5b, Ru SAs‐MoO_3−_ *_x_*/NF presents excellent activity with a relatively low overpotential of 36 mV at the current density of 10 mA cm^−2^, which is close to that of commercial Pt/C on NF (30 mV @ 10 mA cm^−2^). The activity improvement originated from Ru single atoms and amorphous feature can be distinctly concluded as compared to the Ru SAs‐MoO_3_/NF, MoO_3−_ *_x_*/NF and MoO_3_/NF catalysts, which needs 57, 192, and 213 mV, respectively, to reach the same current density. Impressively, the HER activity of Ru SAs‐MoO_3−_ *_x_*/NF even surpasses the Pt/C catalyst at the larger current density above 80 mA cm^−2^. Notably, the outstanding HER activity of Ru SAs‐MoO_3−_ *_x_*/NF outperforms most of the lately reported catalysts (Table S4, Supporting Information).

As shown in Figure 5c, the faster HER kinetics of Ru SAs‐MoO_3−_ *_x_*/NF can be reflected by the small Tafel slope of 41.3 mV dec^−1^, slightly inferior to Pt/C on NF (36.9 mV dec^−1^) but significantly better than Ru SAs‐MoO_3_/NF (51.9 mV dec^−1^), MoO_3−_ *_x_*/NF (111.5 mV dec^−1^), MoO_3_/NF (112.2 mV dec^−1^), and bare NF (152.7 mV dec^−1^). The improved HER performance of Ru SAs‐MoO_3−_ *_x_*/NF can also be proved by the Nyquist plots (Figure 5d; and Figure S18, Supporting Information), validating faster electron transport kinetics. Moreover, the *C*_dl_ and ECSA values are displayed in Table S2 (Supporting Information), indicating the highest ECSA value and the most exposed active sites for Ru SAs‐MoO_3−_ *_x_*/NF. Furthermore, the ECSA‐normalized LSV curves demonstrated that Ru SAs‐MoO_3−_ *_x_*/NF still possesses the best intrinsic activity toward HER among catalysts (Figure S12b, Supporting Information). As exhibited in Figure S13f (Supporting Information), the TOF values were also calculated to evaluate the intrinsic activity of catalysts. For Ru SAs‐MoO_3−_ *_x_*/NF, it owns the highest TOF value (0.47 s^−1^) at the overpotential of 100 mV among all samples, corroborating the advantages of Ru SAs‐MoO_3−_ *_x_*/NF as a better HER catalyst.

The stability of Ru SAs‐MoO_3−_ *_x_*/NF for HER was evaluated by CV cycles and *i*–*t* testing. As shown in Figure 5e, almost no decay can be found after 20 000 CV cycles. Similarly, the activity of Ru SAs‐MoO_3−_ *_x_*/NF preserves stable during a 50 h chronoamperometric test at the constant current density of 50 mA cm^−2^ (Figure 5f). In addition, the FESEM and XPS analysis results (Figure S19, Supporting Information) further confirm the remarkable stability of Ru SAs‐MoO_3−_ *_x_*/NF during the long‐term HER process. Undoubtedly, all the above results illustrate the excellent HER stability of Ru SAs‐MoO_3−_ *_x_*/NF in alkaline media.

### 2.4 Water and Seawater Splitting Performance Evaluation

**Figure 6:**
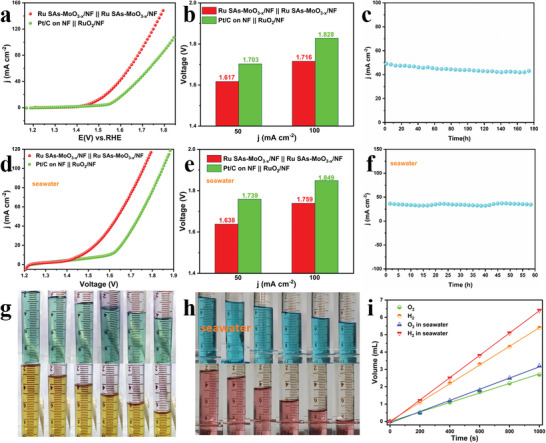
a) Polarization curves of the OWS devices with Ru SAs‐MoO_3−_ *_x_*/NF || Ru SAs‐MoO_3−_ *_x_*/NF couple and Pt/C on NF || RuO_2_ on NF couple. b) Corresponding voltage at *j* = 50 and 100 mA cm^−2^. c) Stability test of Ru SAs‐MoO_3−_ *_x_*/NF || Ru SAs‐MoO_3−_ *_x_*/NF couple in conventional alkaline media. d) Polarization curves of Ru SAs‐MoO_3−_ *_x_*/NF || Ru SAs‐MoO_3−_ *_x_*/NF couple and commercial catalyst couple toward overall water splitting in alkaline seawater media. e) Corresponding voltage at *j* = 50 and 100 mA cm^−2^ in alkaline seawater media. f) Stability test of Ru SAs‐MoO_3−_ *_x_*/NF || Ru SAs‐MoO_3−_ *_x_*/NF couple in alkaline seawater media. Digital photographs of collected H_2_ and O_2_ at different time in (g) 1 m KOH and h) alkaline seawater media. i) Gas volume of collected H_2_ and O_2_ versus time in 1 m KOH and alkaline seawater media.

According to the above experimental results, Ru SAs‐MoO_3−_ *_x_*/NF electrode can serve as a bifunctional electrocatalyst for OER and HER in alkaline media. Consequently, a two‐electrode configuration with the conventional 1 m KOH electrolyte was first assembled by employing Ru SAs‐MoO_3−_ *_x_*/NF as both anode and cathode. As depicted in **Figure** **6**a,b, Ru SAs‐MoO_3−_ *_x_*/NF || Ru SAs‐MoO_3−_ *_x_*/NF couple exhibits prominent overall water splitting (OWS) performance, which can drive the current densities of 10, 50, and 100 mA cm^−2^ at the low cell voltage of 1.487, 1.617, and 1.761 V, respectively. Such performance is even superior to commercial Pt/C on NF || RuO_2_ on NF couple (1.573, 1.703, and 1.828 V @ 10, 50, and 100 mA cm^−2^) and many lately reported similar bifunctional electrocatalysts (Table S5, Supporting Information). Besides, the performance of Ru Sas‐MoO_3_/NF and MoO_3−_ *_x_*/NF bifunctional catalysts was also measured. Figure S20 (Supporting Information) confirms the pivotal role of amorphous structures and Ru single atoms in boosting the water splitting activity. Furthermore, Ru SAs‐MoO_3−_ *_x_*/NF || Ru SAs‐MoO_3−_ *_x_*/NF shows excellent long‐term stability with the almost unattenuated current density after 3000 CV cycles (Figure S21, Supporting Information) and 180 h chronoamperometric test under 50 mA cm^−2^ with activity loss of 0.04 mA cm^−2^ h^−1^ (Figure 6c).

Furthermore, the electrochemical performance of Ru SAs‐MoO_3−_ *_x_*/NF in alkaline seawater electrolytes was evaluated to alleviate the dependence on freshwater resources. The OER and HER performance of Ru SAs‐MoO_3−_ *_x_*/NF and commercial catalysts was first tested in alkaline seawater media. As displayed in Figure S22a (Supporting Information), there are only slight changes in the HER performance of Ru SAs‐MoO_3−_ *_x_*/NF and Pt/C on NF as compared to the performance in alkaline media, which need low overpotentials of 43 and 35 mV to drive the current density of 10 mA cm^−2^, respectively. With respect to OER, Figure S22b (Supporting Information) indicates that the activity of Ru SAs‐MoO_3−_ *_x_*/NF (230 mV @ 10 mA cm^−2^, 321 mV @ 100 mA cm^−2^) is still much better than commercial RuO_2_/NF (291 mV @ 10 mA cm^−2^, 412 mV @ 100 mA cm^−2^). In addition, the slight performance attenuation confirms the outstanding long‐term durability of Ru SAs‐MoO_3−_ *_x_*/NF toward OER and HER (Figure S23, Supporting Information) in alkaline seawater media. As for overall seawater splitting, Ru SAs‐MoO_3−_ *_x_*/NF || Ru SAs‐MoO_3−_ *_x_*/NF couple merely needs cell voltages of 1.638 and 1.759 V to deliver the current densities of 50 and 100 mA cm^−2^, respectively, while Pt/C on NF || RuO_2_ on NF couple delivers the same current density at larger cell voltages of 1.739 and 1.849 V (Figure 6d,e). When compared with other reported catalysts, such a bifunctional electrode is still competitive (Tables S6 and S7, Supporting Information). In addition, the high stability of Ru SAs‐MoO_3−_ *_x_*/NF || Ru SAs‐MoO_3−_ *_x_*/NF in alkaline seawater media can be demonstrated by a 60 h *i*–*t* testing (Figure 6f) and the CV cycling test (Figure S24, Supporting Information).

As shown in Figure S25 (Supporting Information), the hydrogen and oxygen gases generated on Ru SAs‐MoO_3−_ *_x_*/NF electrodes can be observed by Videos S1 and S2 (Supporting Information), and were collected through a water drainage method using a H‐type electrolytic cell where an anion‐exchange membrane was used as the separator. The volume changes during 1000 s electrolysis in 1 m KOH media and 1 m KOH seawater media are displayed in Figure 6g–i. The emissions of hydrogen and oxygen are 5.4: 2.7 mL and 6.4: 3.2 mL, respectively, in 1 m KOH and alkaline seawater media, indicating a volume ratio of 2:1 and almost 100% Faradic efficiency (98.3% and 99.6%) for overall water and seawater splitting. All the above results demonstrate the practical application potential of Ru SAs‐MoO_3−_ *_x_*/NF as excellent bifunctional electrocatalyst toward water and seawater splitting.

## 3 Conclusion

In summary, a bifunctional water and seawater splitting catalyst Ru SAs‐MoO_3−_ *_x_*/NF was designed and constructed. The 3D flower‐like morphology and amorphous structure afford large surface area and more exposed active sites for electrocatalytic reactions. Benefitting from the oxygen vacancies of MoO_3−_ *_x_*, Ru single atoms are uniformly fixed on the amorphous substrate, thus optimizing the bifunctional performance of the catalyst for OER and HER. Specifically, the as‐prepared Ru SAs‐MoO_3−_ *_x_*/NF electrode possesses excellent OER, HER, and overall water splitting activity and robust stability in both alkaline and seawater media. Therefore, our work may shed new light on an approach to rationally designing and developing catalysts with outstanding performance for freshwater and seawater splitting.

## Conflict of Interest

The authors declare no conflict of interest.

## Supporting information

Supporting Information

Supplemental Video 1

Supplemental Video 2

## Data Availability

Research data are not shared.
