# Supplementary material for: Flower‐Like Amorphous MoO3− x Stabilized Ru Single Atoms for Efficient Overall Water/Seawater Splitting
Source: Adv Sci (Weinh). 2023 Apr 24;10(18):2300342. doi: 10.1002/advs.202300342 (PMC10288252; doi:10.1002/advs.202300342)
Supplement: Supplementary file 1 — Supporting Information [file ADVS-10-2300342-s002.pdf]

ADVANCED SCIENCE
Open Access

Supporting Information

for *Adv. Sci.*, DOI 10.1002/advs.202300342

Flower-Like Amorphous MoO_{3-x} Stabilized Ru Single Atoms for Efficient Overall Water/Seawater Splitting

Dong Feng, Pengyan Wang, Rui Qin, Wenjie Shi, Lei Gong, Jiawei Zhu, Qianli Ma, Lei Chen, Jun Yu, Suli Liu and Shichun Mu**

Supporting Information

Flower-like Amorphous MoO_{3-x} Stabilized Ru Single Atoms for Efficient Overall Water/Seawater Splitting

Dong Feng, Pengyan Wang, Rui Qin, Wenjie Shi, Lei Gong, Jiawei Zhu, Qianli Ma, Lei Chen, Jun Yu, Suli Liu, Shichun Mu**

D. Feng, P. Wang, R. Qin, W. Shi, L. Gong, J. Zhu, Q. Ma, L. Chen, J. Yu, Prof. S. Mu

State Key Laboratory of Advanced Technology for Materials Synthesis and Processing, Wuhan University of Technology, Wuhan, 430070, China

D. Feng, Prof. S. Mu

Foshan Xianhu Laboratory of the Advanced Energy Science and Technology Guangdong Laboratory, Xianhu Hydrogen Valley, Foshan, 528200, China.

Prof. S. Liu

Key Laboratory of Advanced Functional Materials of Nanjing, Nanjing Xiaozhuang University, Nanjing, 211171, China.

Corresponding author, E-mail: msc@whut.edu.cn, niuniu_410@126.com

Experimental Details

Materials and Reagents

Ammonium molybdate tetrahydrate ($(NH_4)_6Mo_7O_{24}\cdot4H_2O$), Sodium dodecyl sulfate（SDS）, absolute ethanol, hydrochloric acid (HCl) and potassium hydroxide (KOH) were purchased from Sinopharm Chemical Reagent Co., Ltd. Ruthenium(III) chloride hydrate ($RuCl_3\cdot xH_2O$) was purchased from Shaanxi Kaida Chemical Engineering Co., Ltd. Commercial Pt/C catalyst (20%), RuO_2 catalyst and Nafion solution (5%) were bought from Sigma-Aldrich Chemical Reagent Co., Ltd. The above regents were analytical reagents and used without further purification. Deionized Mini-Q water was employed as solvent. And the seawater was collected from the Yellow Sea of China, and artificially filtered for further use. The nickel foam (NF) was purchased from Lyrun Material Co., Ltd, and the thickness of NF was 1 mm.

Synthesis of Ru SAs-MoO_{3-x}/NF

A piece of NF (2.3*3 cm) was first clean by sonication in 10% HCl for several minutes, then it was carefully sonicated in ethanol and deionized water for 30 min to fully eliminate the oxides on the surface. The pre-treated NF was placed in a 30 mL aqueous solution containing 741.5 mg $(NH_4)_6Mo_7O_{24}\cdot4H_2O$ and 865 mg SDS, which was then transferred into a Teflon-lined stainless steel autoclave. The precursor can be obtained by hydrothermal reaction at 100 ℃ for 18 h, followed by rinsing with water

and vacuum drying at 60 ℃ for 10 h. After that, the precursor was immersed into 5 mL of $RuCl_3·xH_2O$ ethanol solution (20 mg/mL) at room temperature for 2 h, following by vacuum drying at 60 ℃ for 10 h. After that, the precursor was annealed at 350 ℃ for 2 h in the tube furnace. Finally, the Ru SAs-MoO_{3-x}/NF can be obtained by cooling to room temperature.

Synthesis of comparative samples

The preparation of Ru SAs-MoO_3/NF was similar to the Ru SAs-MoO_{3-x}/NF except for the different annealing condition, which was heated at 450 ℃ for 2 h. The MoO_{3-x}/NF and MoO_3/NF were obtained in the same way as the Ru SAs-MoO_{3-x}/NF and Ru SAs-MoO_3/NF except for the absence of Ru, respectively.

Material Characterization

Powder X-ray diffraction (XRD) patterns were collected on Bruker D8 Advance from 20˚ to 80˚. The surface morphology of samples was observed by Field emission scanning electron microscopy (FESEM Zeiss Ultra Plus). The further structural information was unveiled with high-resolution transmission electron microscope (HRTEM, JEM-2100F) and double spherical aberration-corrected scanning transmission electron microscope (AC-STEM, Titan Cubed Themis G2 300). X-ray photoelectron spectrometer (Thermo Scientific K-Alpha) was employed to investigate the surface elemental composition and valence bond structure. The ICP-AES analysis obtained on Optima Prodigy 7(LEEMAN LABS Ltd., USA).

XAFS analysis

Data reduction, data analysis, and EXAFS fitting were performed with the Athena and Artemis programs of the Demeter data analysis packages that utilizes the FEFF6 program to fit the EXAFS data. The energy calibration of the sample was conducted through standard Mo foil and Ru foil, which was simultaneously measured as a reference. A linear function was subtracted from the pre-edge region, then the edge jump was normalized using Athena software. The $\chi(k)$ data were isolated by subtracting a smooth, third-order polynomial approximating the absorption background of an isolated atom. The $k3$-weighted $\chi(k)$ data were Fourier transformed after applying a Kaiser-Bessel window function (Δk = 1.0). For EXAFS modeling, the global amplitude EXAFS (CN, R, σ^2 and ΔE_0) was obtained by using Artemis software to nonlinear fit the EXAFS equation with Fourier transform data in R space (least square refinement). Then, to determine the coordination numbers (*CNs*) in the Mo/Ru-O/Ru scattering path in sample, EXAFS of the Mo foil and Ru foil was fitted and the obtained amplitude reduction factor S_0^2 values (0.854 and 0.880) were set in the EXAFS analysis.

Electrochemical Measurements

All electrochemical measurements were performed in a conventional three-electrode system at room temperature using a CHI 660E electrochemical analyzer (CHI Instruments, Shanghai, China). A graphite rod and Hg/HgO were used

as the counter electrode and the reference electrode, respectively. The as-prepared catalysts with dimensions of 0.5 cm×0.5 cm were directly employed as the working electrodes, and the loading of catalysts is about 3.5 mg cm^{-2}. As for powdery catalysts (RuO_2 and Pt/C), the working electrodes were prepared by sonicating the mixture containing 5 mg powder RuO_2 or Pt/C catalysts, 440 μL of isopropanol, 50 μL of water and 10 μL of 5 wt % Nafion for 30 min. The catalyst loading was 3.5 mg cm^{-2}, similar to that of Ru SAs-MoO_{3-x}/NF. The electrolytes were 1 M KOH freshwater solution and 1 M KOH seawater solution. To prepare the alkaline seawater media, the collected seawater was first filtered to remove the insoluble impurities. Then the potassium hydroxide was added into the seawater to obtain the 1 M KOH solution. After stirring for 30 min, the solution was filtered again to remove the precipitated substances. The alkaline electrolyte mainly contains cationic K^+, Na^+, anionic OH^-, Cl^-, SO_4^{2-}, as well as other trace ions. The chloride concentration in the alkaline seawater media was measured to be 10960.07 mg/L by the Ion Chromatography test.

In HER and OER characterizations, all the polarization curves were recorded at a scan rate of 5 mV s^{-1}, and the polarization curves were iR-corrected using the equation: $E_{iR\text{-}corrected}$ = E-iR, where E is the original potential, R is the solution resistance, i is the corresponding current, and $E_{iR\text{-}corrected}$ is the iR-corrected potential. Electrochemical impedance spectroscopy (EIS) tests were carried out in a frequency ranging from 0.1 Hz to 100 kHz with AC amplitude of 10 mV. Furthermore, the HER and OER potentials were converted to RHE scale according to the equation: E (vs. RHE) = E (vs. Hg/HgO) + 0.059*pH + 0.098 V. The electrochemical double layer capacitance

(C_{dl}) was determined with typical cyclic voltammetry (CV) measurements at various scan rates (20, 40, 60, 80, 100, 120 mV s^{-1}) in 0 ~ 0.1 V versus Hg/HgO region by the formula of $C_{dl} = \Delta j/2\nu$, where v is the scan rate, Δj is the current density difference. The generated H_2 and O_2 gases during overall water splitting were quantitatively collected by the water drainage method. The electrochemical active surface area (ECSA) was estimated by the obtained C_{dl} according to the formula: ECSA = C_{dl}/C_s, where the C_s means the specific capacitance of an ideal 1 cm^2 flat surface. Here we adopt the general value of 60 μF cm^{-2} for C_s.

As for the two-electrode water splitting performance, the polarization curve without iR compensation was recorded with the as-synthesized bifunctional electrode material as both anode and cathode. The Pt/C||RuO_2 couple (3.5 mg cm^{-2}) was also measured as benchmark. The stability was assessed through CV cycling test and chronoamperometry. The generated H_2 and O_2 were separated in a typical H-style cell with an anion-exchange membrane, and then collected and quantitatively evaluated by the drainage method.

Turnover frequency (TOF) calculations

The TOF (s^{-1}) value was estimated by the following formula:

$$TOF = I/mnF$$

where I is current (A) during the linear sweep voltammetry (LSV) tests in 1 M KOH, n is the number of active sites (mol), F is the Faraday constant (96485, C mol^{-1}), m is the factor (m for hydrogen evolution and oxygen evolution reactions are 2 and 4, respectively).

The number of active sites (mol) was calculated by the following formula:

$$n = Q/2F = It/2F = IV/2Fv$$

where Q is the voltammetric charge, F is the Faraday constant (C mol^{-1}), I stands for the current (A), t is the time (s), V is the voltage (V) and v is the scanning rate (V s^{-1}).

Supplemental results

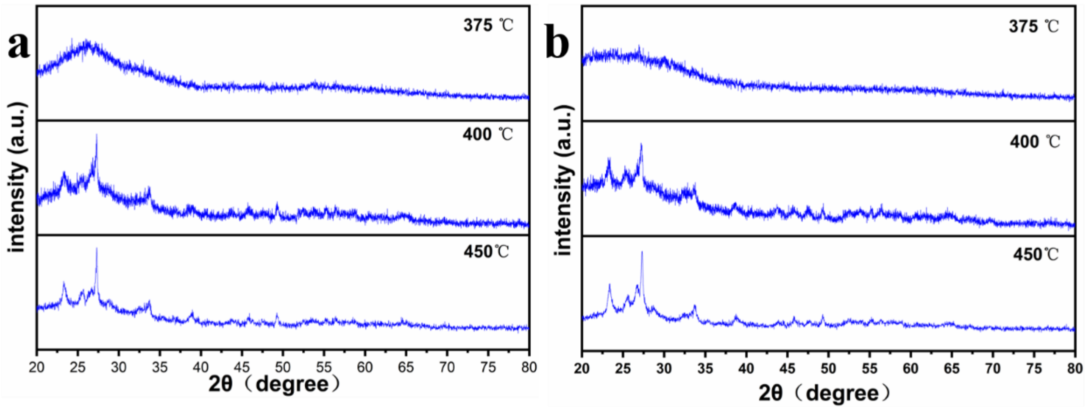

Figure S1. a) XRD patterns of Ru-incorporating samples with different annealing temperatures. b) XRD patterns of samples without Ru incorporation at different annealing temperatures

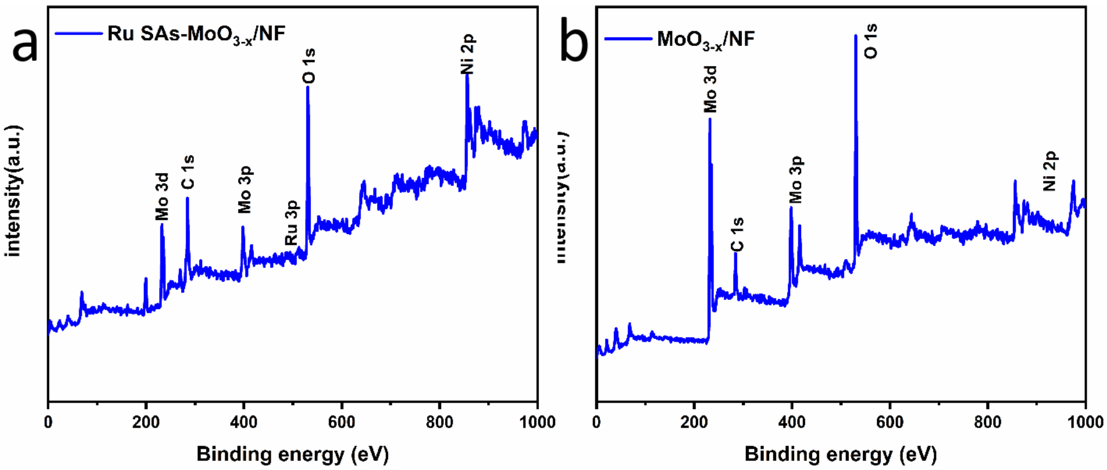

Figure S2. The XPS spectra of a) Ru SAs-MoO_{3-x}/NF and b) MoO_{3-x}/NF

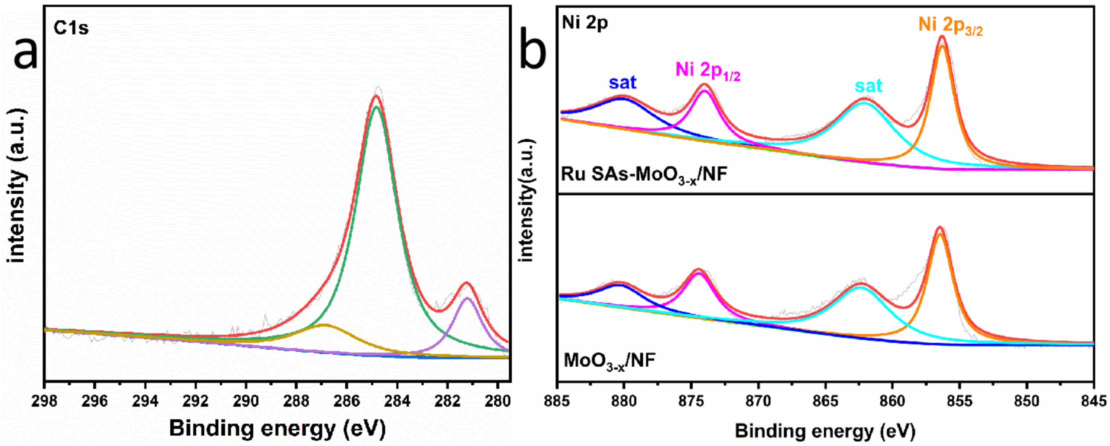

Figure S3. a) C 1s and b) Ni 2p deconvoluted spectra of Ru SAs-MoO_{3-x}/NF.

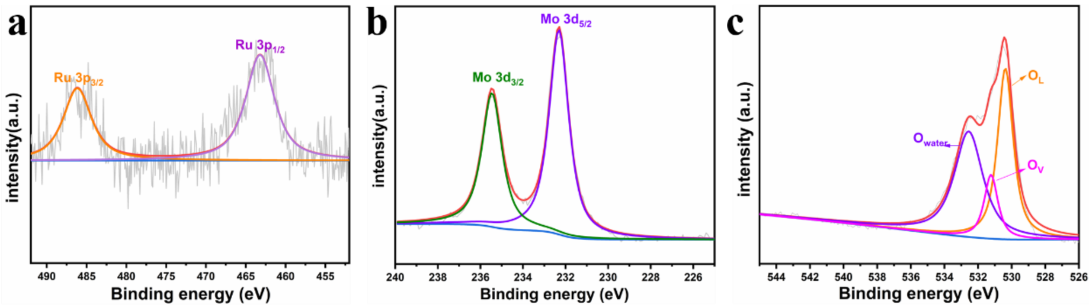

Figure S4. a) Ru 3p, b) Mo 3d and c) O1s deconvoluted spectra of Ru SAs-MoO_3/NF.

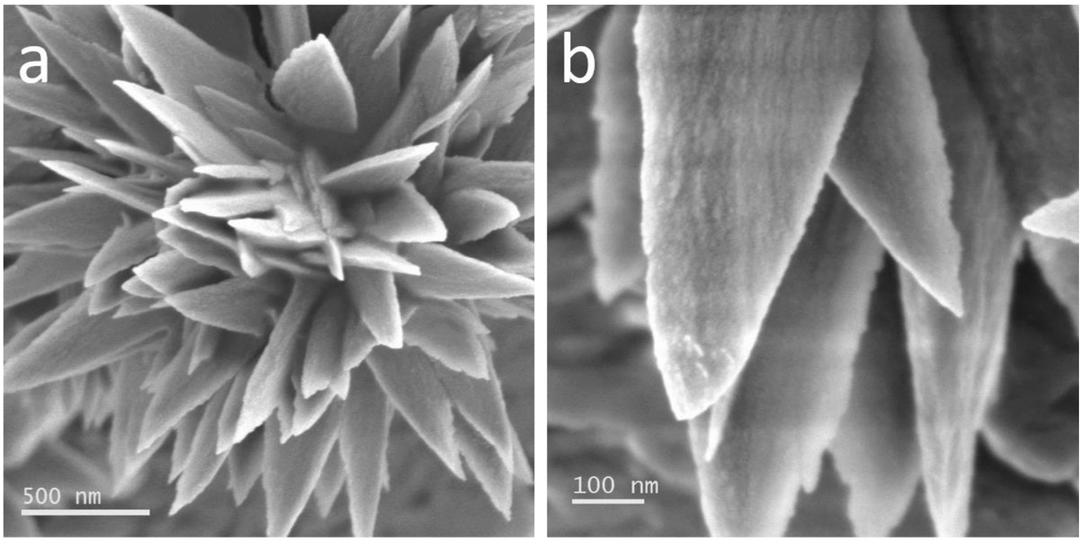

Figure S5. The FESEM images of the precursor.

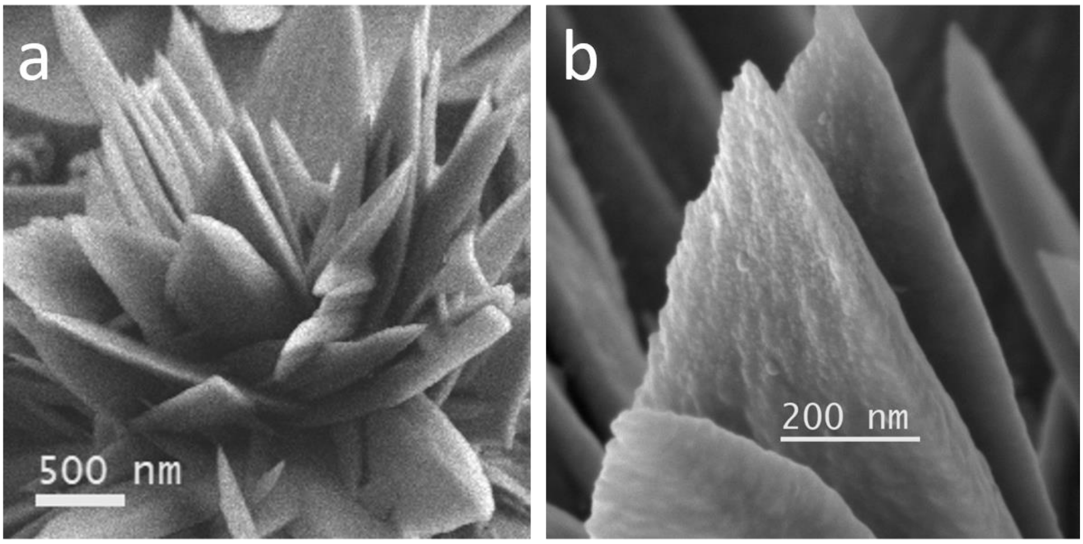

Figure S6. The FESEM images of MoO_{3-x}/NF

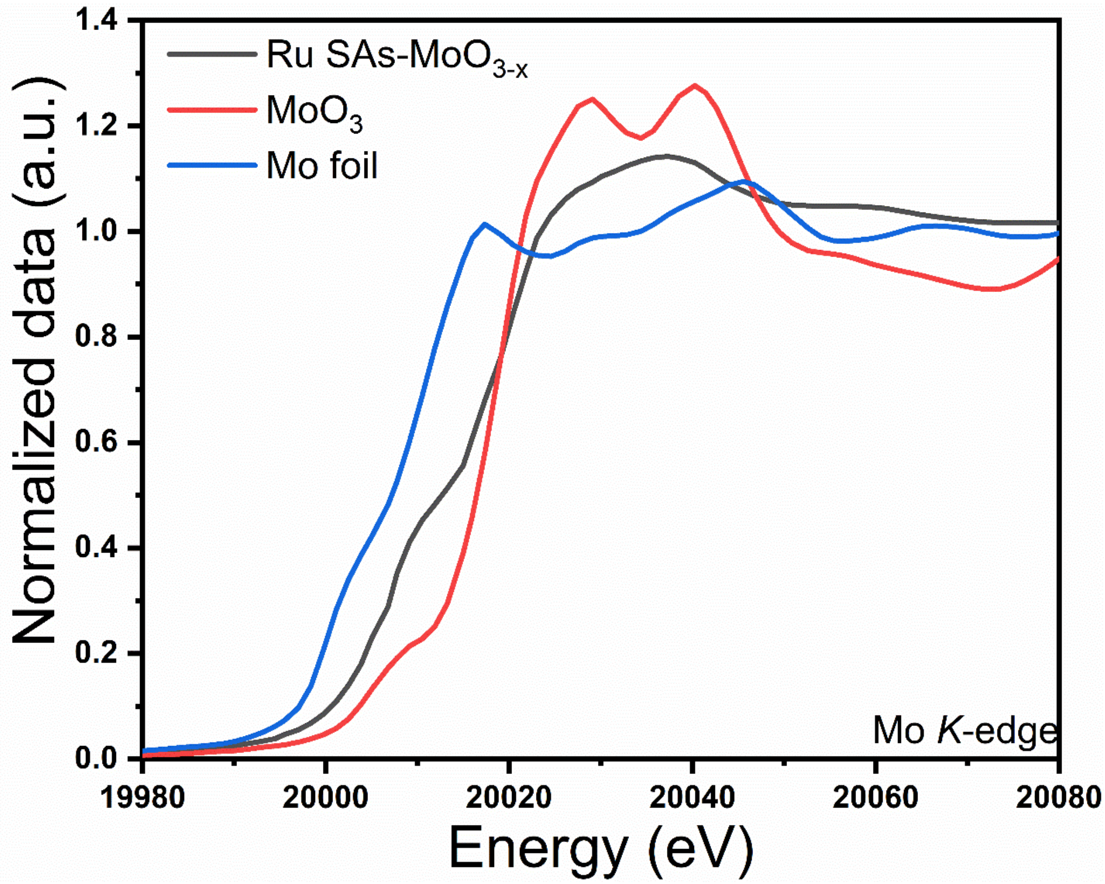

Figure S7. Mo K-edge XANES for Ru SAs-MoO_{3-x}, MoO_3, and Mo foil.

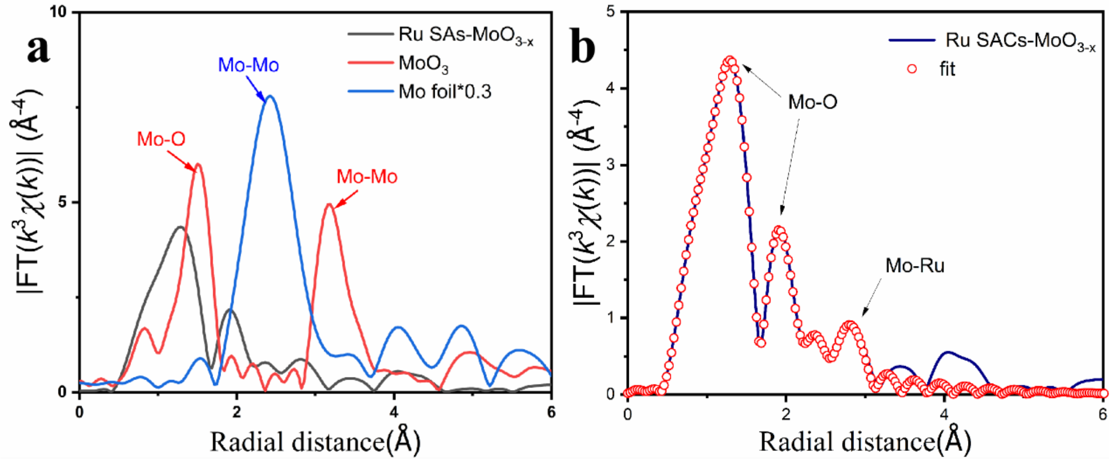

Figure S8. Mo K-edge EXAFS for Ru SAs-MoO_{3-x}, MoO_3, and Mo foil.

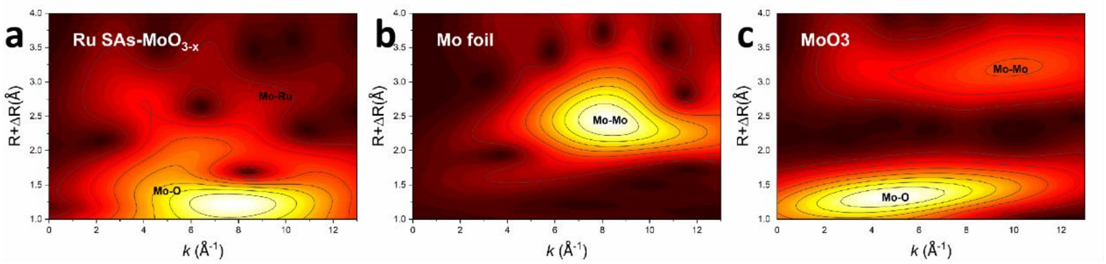

Figure S9. a-c) WT for the k2-weighted EXAFS signal of Ru SAs-MoO_{3-x}, Mo foil, and MoO_3.

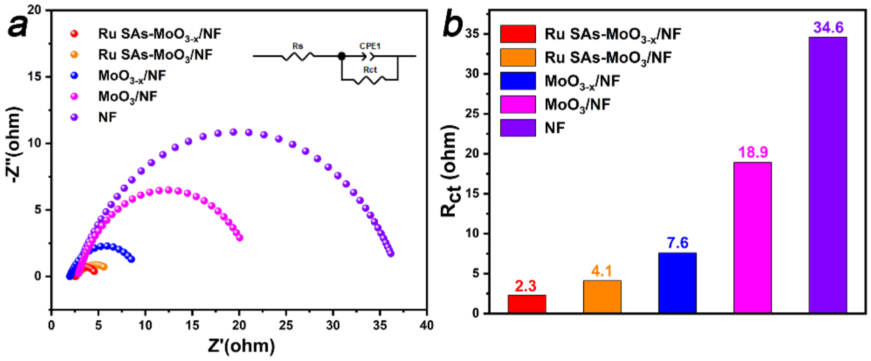

Figure S10. a) Fitting EIS plots with equivalent circuit and b) corresponding R_{ct} values of Ru SAs-MoO_{3-x}/NF, Ru SAs-MoO_3/NF, MoO_{3-x}/NF, MoO_3/NF and bare NF for OER.

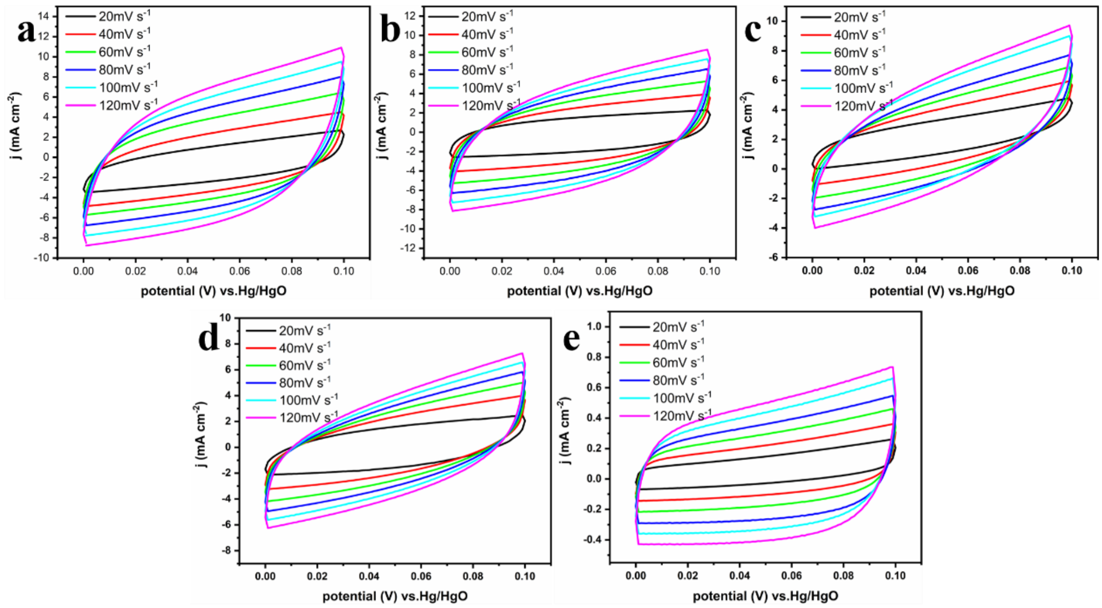

Figure S11. Cyclic voltammetry (CV) curves at different scan rates for a) Ru SAs-MoO_{3-x}/NF, b) Ru SAs-MoO_3/NF, c) MoO_{3-x}/NF, d) MoO_3/NF and (e) bare NF

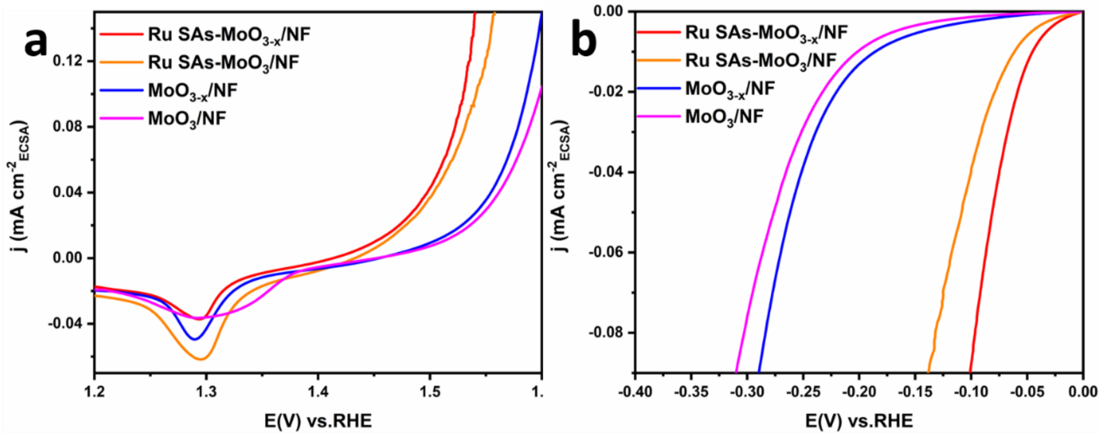

Figure S12. ECSA normalized polarization curves of the as-prepared electrodes toward a) OER and b) HER.

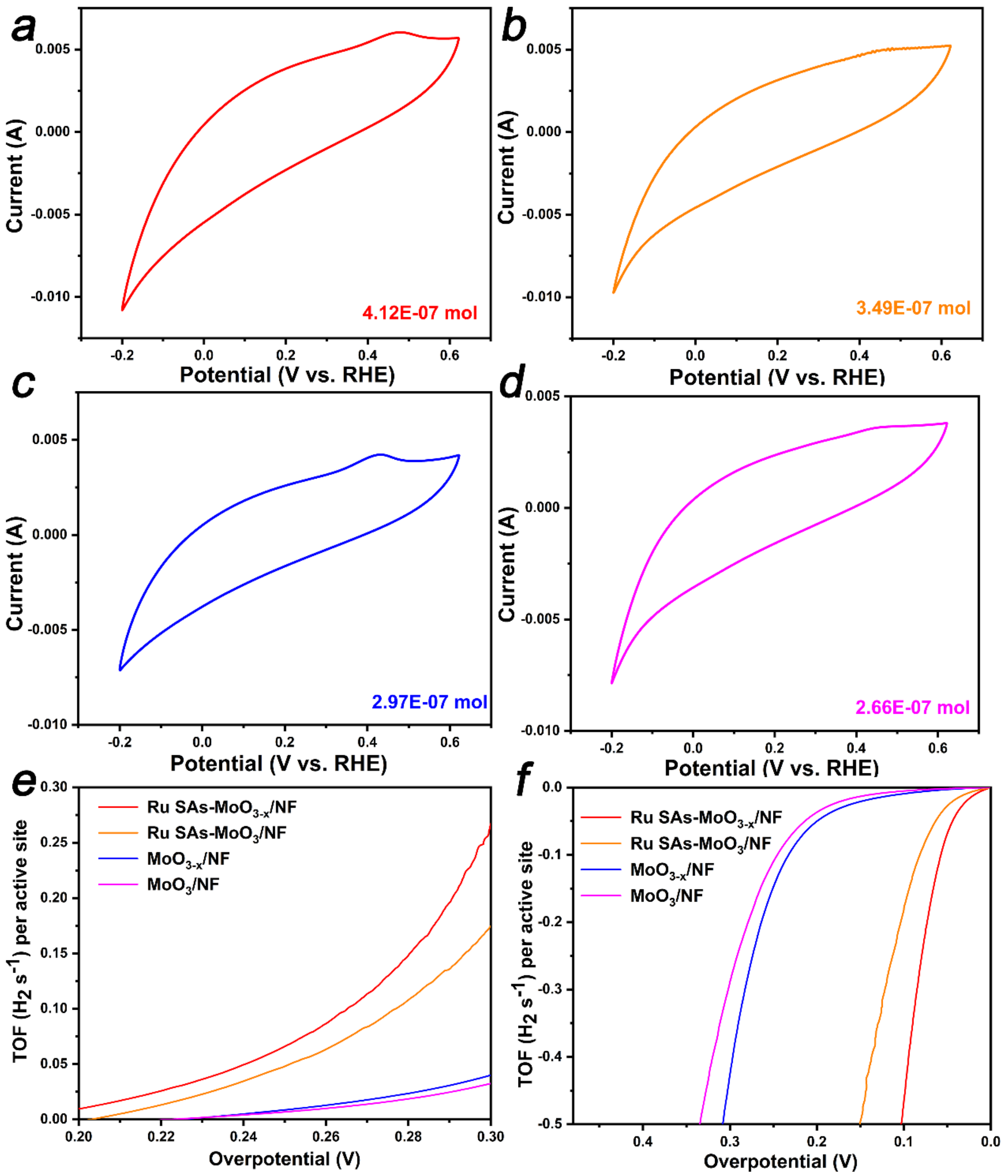

Figure S13. Cyclic voltammogram (CV) curves of (a) Ru SAs-MoO_{3-x}/NF, (b) Ru SAs-MoO_3/NF, (c) MoO_{3-x}/NF, (d) MoO_3/NF electrocatalysts measured in 1 M PBS (pH = 7) electrolyte at a scan rate of 50 mV s^{-1}. Based on the formula of n = IV/2Fv, the number of active sites was determined to be 4.12E-07 mol, 3.49E-07 mol, 2.97E-07 mol and 2.66E-07 mol for Ru SAs-MoO_{3-x}/NF, Ru SAs-MoO_3/NF, MoO_{3-x}/NF, MoO_3/NF, respectively. Calculated (e) O_2 TOF values and (f) H_2 TOF

values for Ru SAs-MoO_{3-x}/NF, Ru SAs-MoO_3/NF, MoO_{3-x}/NF, MoO_3/NF.

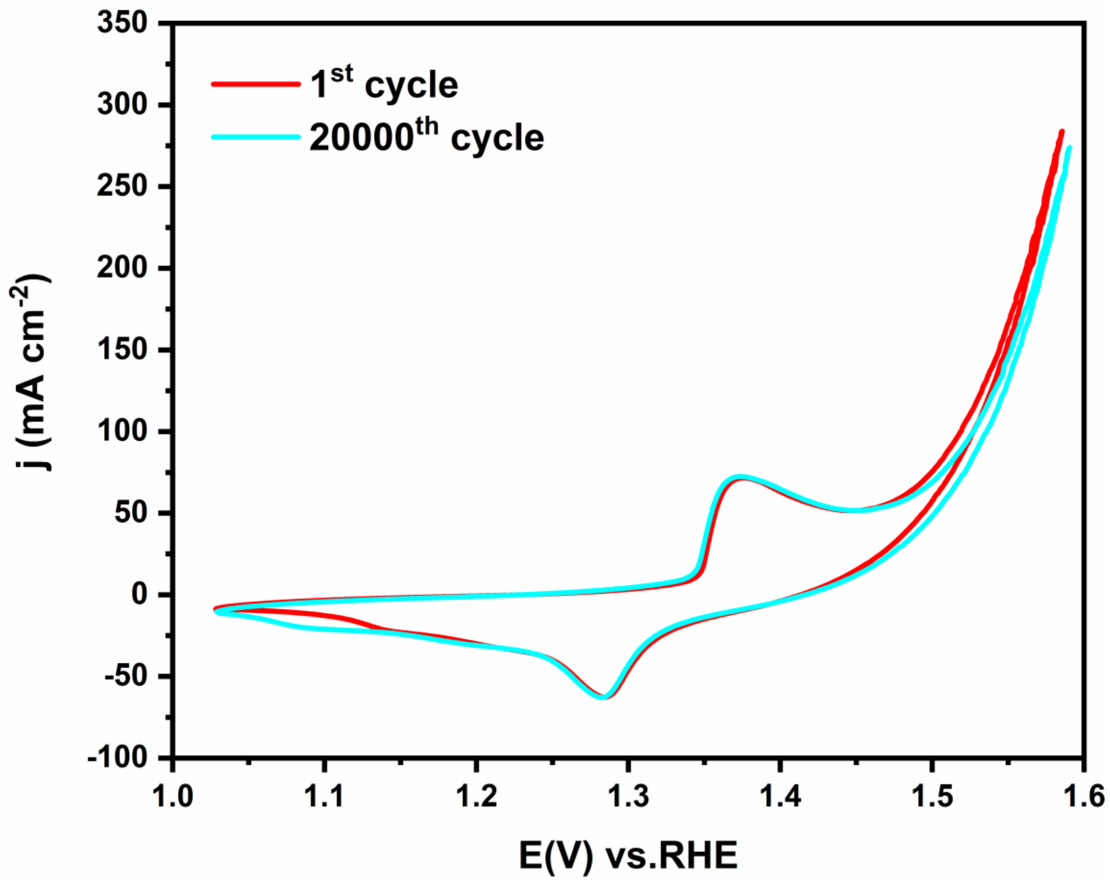

Figure S14. Polarization curves recorded before and after 20000 CV cycles for Ru SAs-MoO_{3-x}/NF. The presence of oxidation peaks at 1.37 V and reduction peaks at 1.29 V in CV curves can be attributed to the conversion between $Ni^{2+}$and Ni^{3+}.

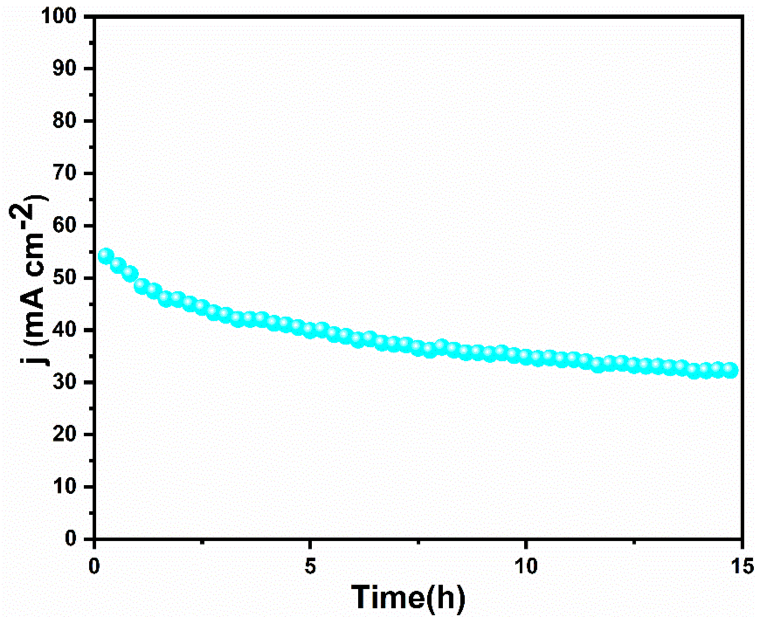

Figure S15. The long-term i-t response test for commercial RuO_2/NF eletrode

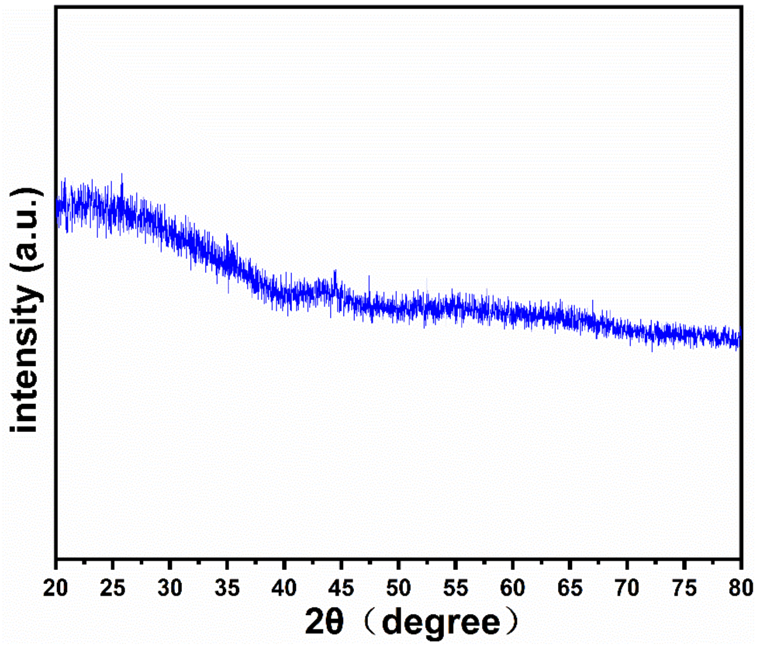

Figure S16. The XRD pattern of Ru SAs-MoO_{3-x}/NF after OER test.

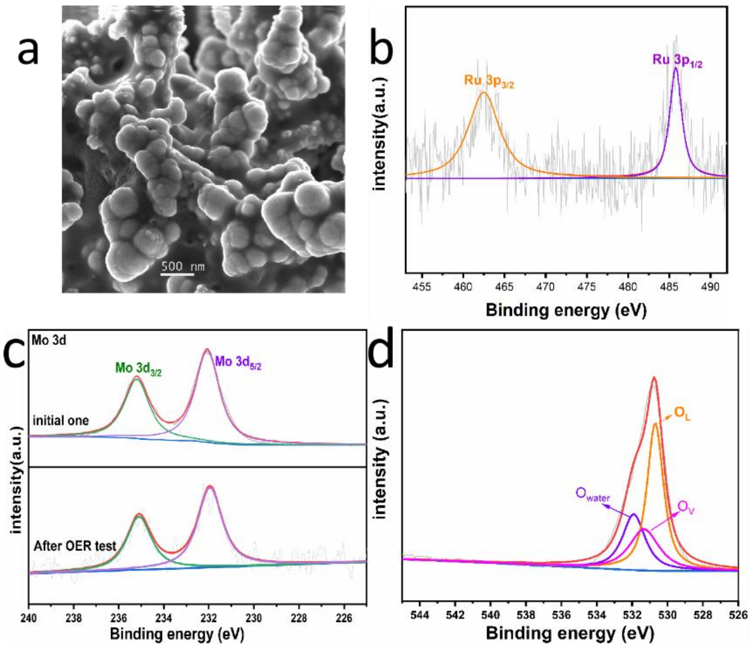

Figure S17. a) The FESEM image of Ru SAs-MoO_{3-x}/NF after OER test. b) Ru 3p and d) O 1s high-resolution XPS spectra of Ru SAs-MoO_{3-x}/NF after OER test. c) Mo 3d high-resolution XPS spectra of Ru SAs-MoO_{3-x}/NF before and after OER test.

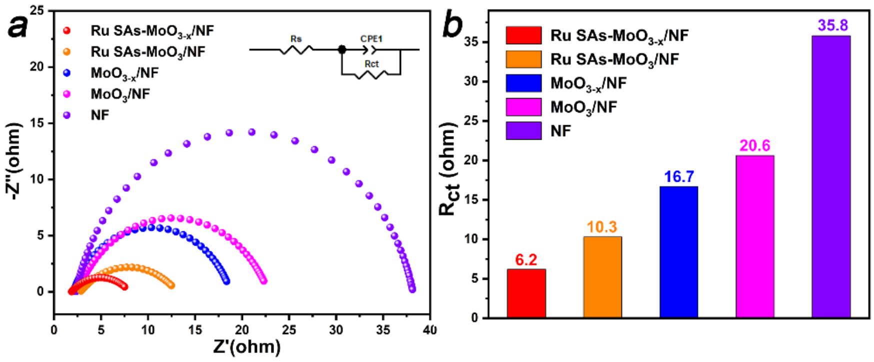

Figure S18. a) Fitting EIS plots with equivalent circuit and b) corresponding R_{ct} values of Ru SAs-MoO_{3-x}/NF, Ru SAs-MoO_3/NF, MoO_{3-x}/NF, MoO_3/NF and bare NF for HER.

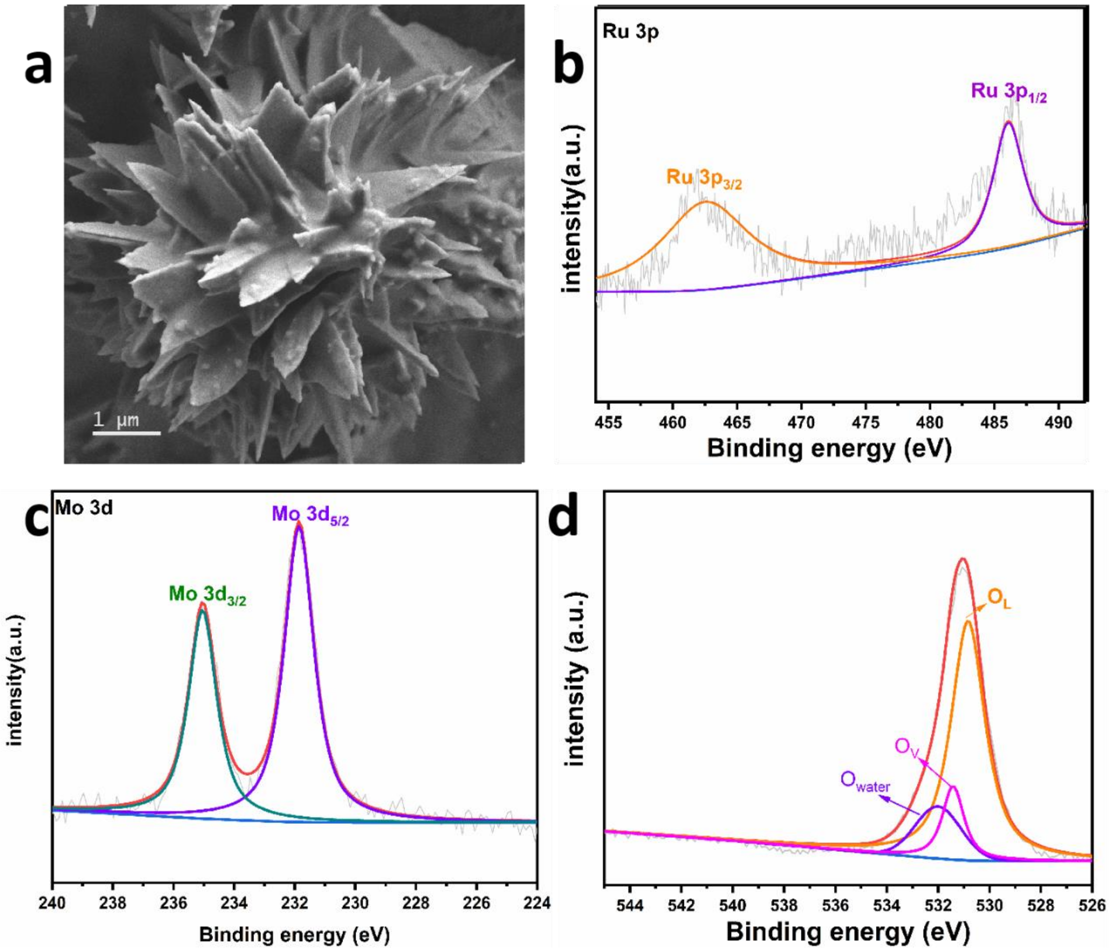

Figure S19. a) The FESEM image of Ru SAs-MoO_{3-x}/NF after HER test. b) Ru 3p c) Mo 3d and d) O 1s high-resolution XPS spectra of Ru SAs-MoO_{3-x}/NF after HER test.

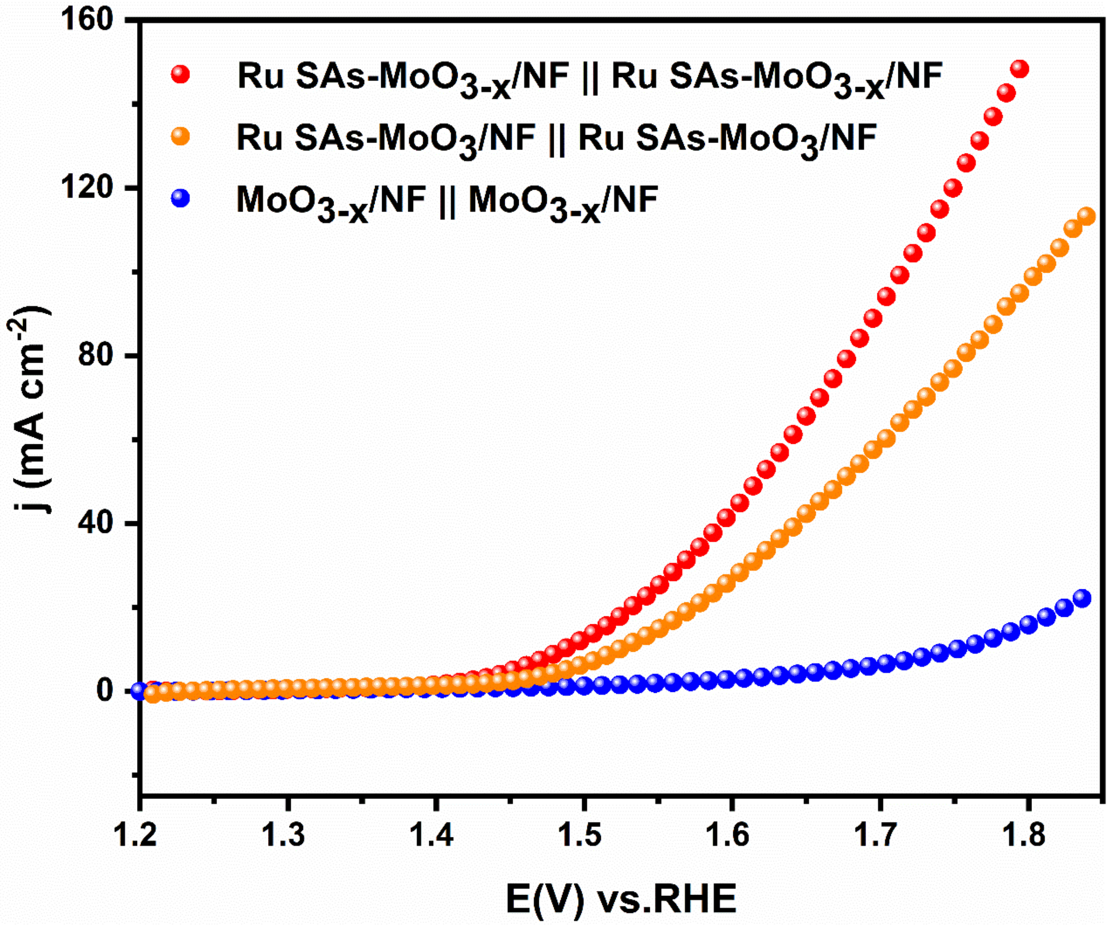

Figure S20. Polarization curves of the OWS devices with Ru SAs-MoO_{3-x}/NF || Ru SAs-MoO_{3-x}/NF couple, Ru SAs-MoO_3/NF || Ru SAs-MoO_3/NF couple and MoO_{3-x}/NF || MoO_{3-x}/NF couple

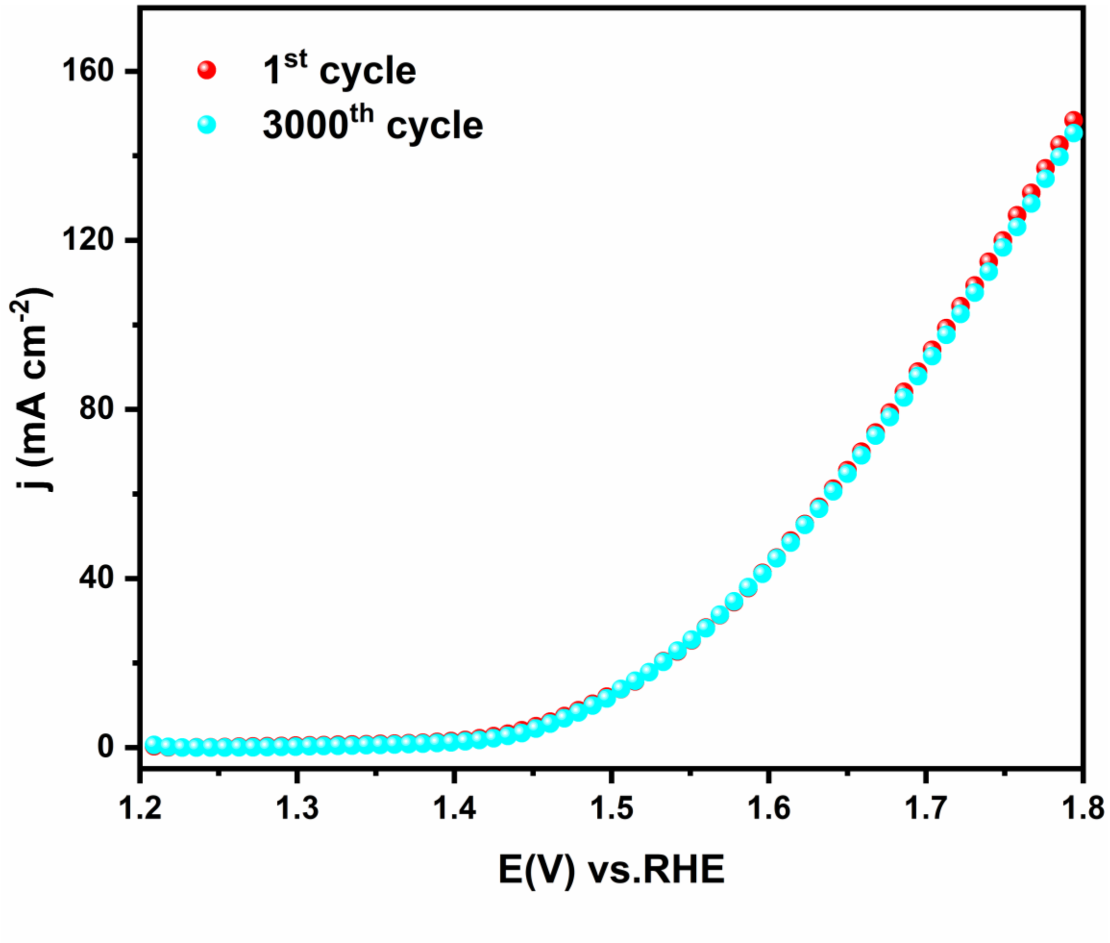

Figure S21. Polarization curves before and after 3000 CV cycles for Ru SAs-MoO_{3-x}/NF in 1 M KOH.

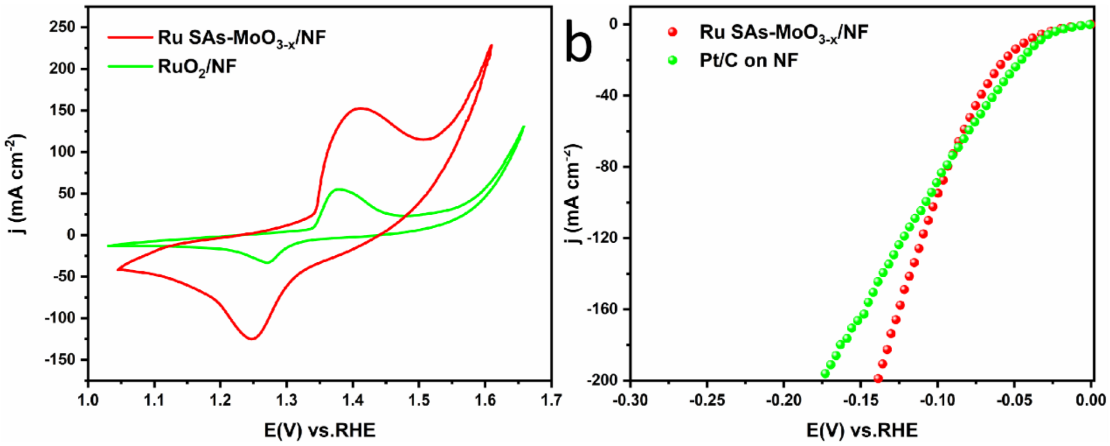

Figure S22. a) OER and b) HER polarization curves with iR-compensation for Ru SAs-MoO_{3-x}/NF and the commercial catalysts in alkaline seawater media recorded at 5 mV s^{-1}.

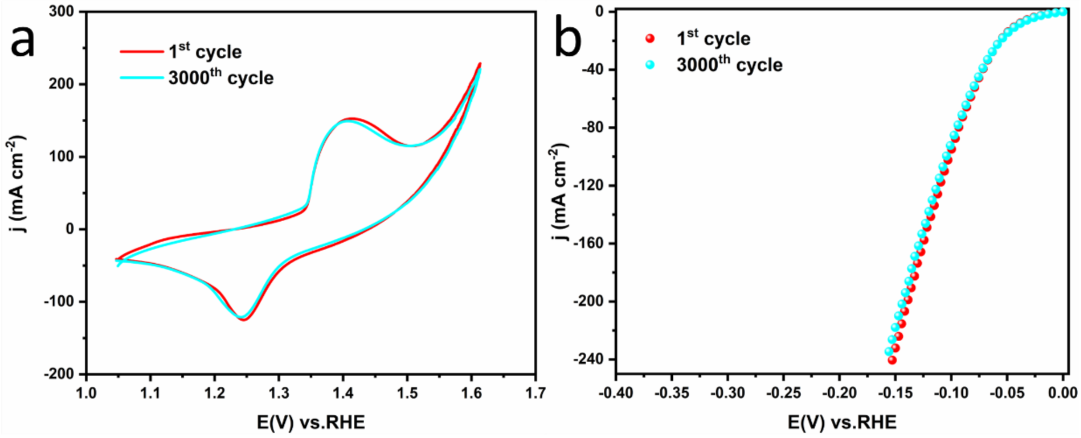

Figure S23. Stability test of Ru SAs-MoO_{3-x}/NF toward a) OER and b) HER in alkaline seawater media.

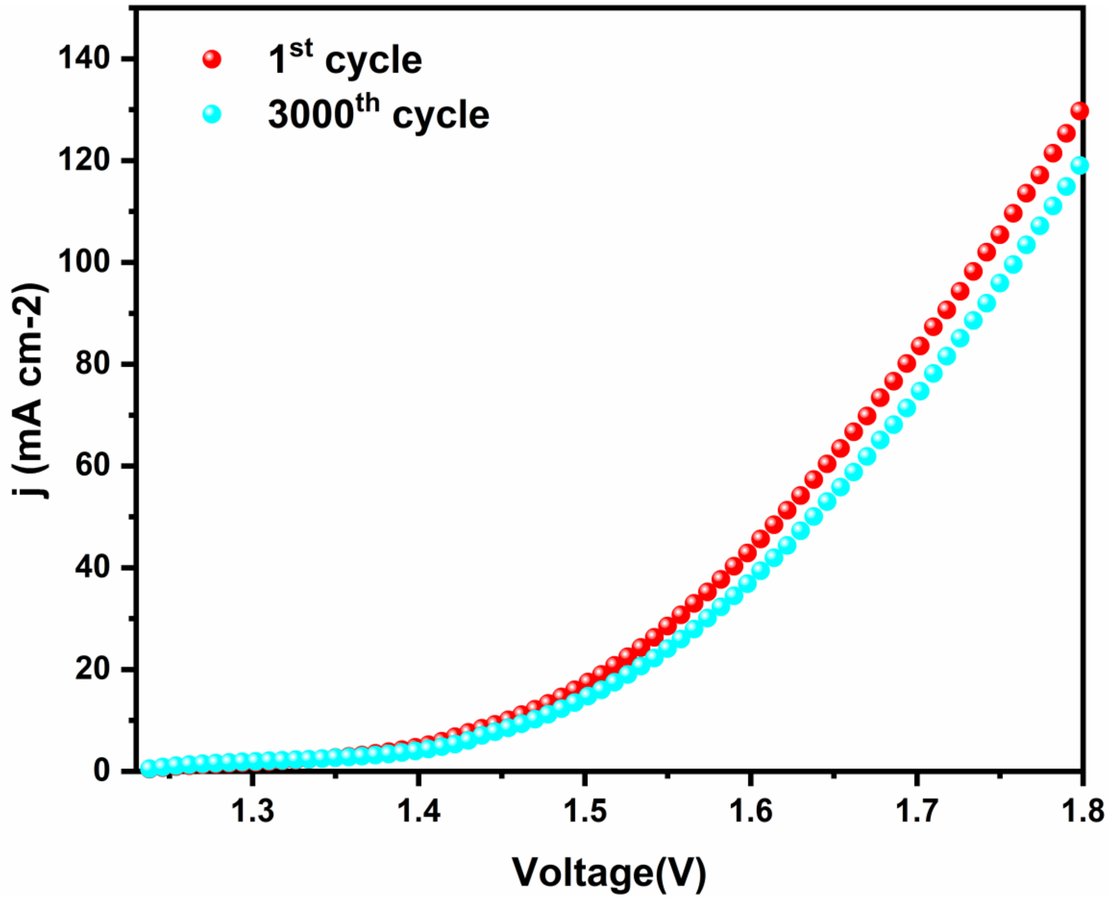

Figure S24. Polarization curves before and after 3000 CV cycles for Ru SAs-MoO_{3-x}/NF in alkaline seawater media.

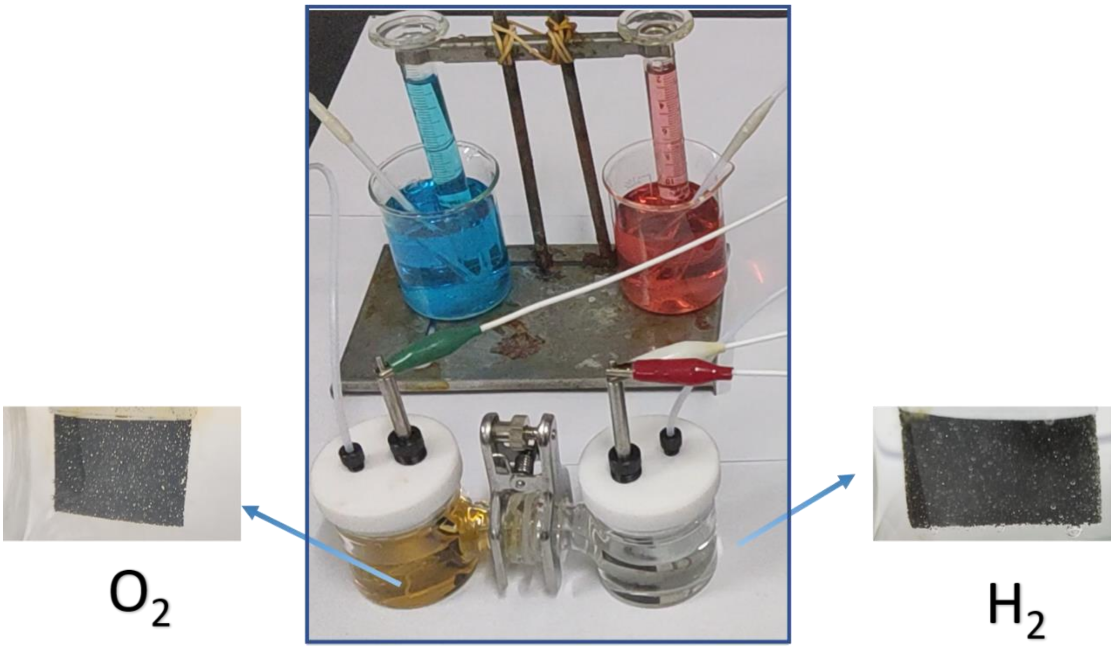

Figure S25. Gases collected device of water splitting and the enlarged diagram of gases evolved on the electrode surface.

Table S1. EXAFS fitting parameters at the M *K*-edge for various samples.

Sample	Shell	CN^a	R(Å)^b	σ^2(Å^2)^c	ΔE_0(eV)^d	R factor
Mo *K*-edge (S_0^2=0.854)						
Mo foil	Mo-Mo	8*	2.716±0.004	0.0034±0.0006	-5.5±0.8	0.0032
	Mo-Mo	6*	3.131±0.005			
MoO3	Mo-O	6.1±0.5	1.953±0.005	0.0031±0.0005	-2.9±1.3	0.0031
	Mo-Mo	4.0±0.6	3.384±0.003	0.0065±0.0029		
Ru SAs-Mo O_{3-x}	Mo-O	2.8±0.3	1.704±0.013	0.0030±0.0023	3.6±5.5	0.0042
	Mo-O	1.5±0.5	2.281±0.018			
	Mo-Ru	1.3±0.4	3.096±0.019	0.0141±0.0027	-2.3±0.4	
Ru *K*-edge (S_0^2=0.880)						
Ru foil	Ru-Ru	12*	2.674±0.004	0.0030±0.0005	-6.1±0.9	0.0090
RuO2	Ru-O	5.8±0.3	1.958±0.008	0.0044±0.0011	0.2±1.5	0.0058
	Ru-Ru	4.6±0.5	3.382±0.015	0.0155±0.0020		
Ru SAs-Mo O_{3-x}	Ru-O	5.0±0.1	2.021±0.016	0.0076±0.0024	2.2±2.7	0.0029

^{a}CN, coordination number; ^{b}R, the distance to the neighboring atom; $^{c}\sigma^2$, the Mean Square Relative Displacement (MSRD); $^{d}\Delta E_0$, inner potential correction; R factor indicates the goodness of the fit. $S0^2$ was fixed to 0.854 and 0.880, according to the experimental EXAFS fit of Mo foil and Ru foil by fixing CN as the known crystallographic value. * This value was fixed during EXAFS fitting, based on the known structure of Mo and Ru. Fitting range: $3.0 \leq k$ (/Å) ≤ 12.0 and $1.0 \leq R$ (Å) ≤ 3.0 (Mo foil); $3.0 \leq k$ (/Å) ≤ 13.0 and $1.0 \leq R$ (Å) ≤ 4.0 (MoRu-Mo); $3.0 \leq k$ (/Å) ≤ 13.2 and $1.0 \leq R$ (Å) ≤ 3.0 (Ru foil), $3.0 \leq k$ (/Å) ≤ 11.0 and $1.0 \leq R$ (Å) ≤ 2.5 (MoRu-Ru). A reasonable range of EXAFS fitting parameters: $0.700 < S_0^2 < 1.000$; $CN > 0$; $\sigma^2 > 0$ Å^2; $|\Delta E_0| < 10$ eV; R factor < 0.02.

Table S2. C_{dl} and ECSA values of the as-prepared catalysts in the region of 0~0.1 V versus Hg/HgO.

Catalyst	C_{dl} (mF cm^{-2})	ECSA (cm^2)
Ru SAs-MoO_{3-x}/NF	101.06	1684.3
Ru SAs-MoO_3/NF	73.44	1224.0
MoO_{3-x}/NF	52.63	877.2
MoO_3/NF	46.76	779.3
Bare NF	7.33	122.2

Table S3. Comparison of OER performance for Ru SAs-MoO_{3-x}/NF with other reported electrocatalysts in 1 M KOH.

Catalysts	η@j (mV @ mA cm^{-2})	Tafel slope (mV dec^{-1})	Loading of catalyst (mg/cm^2)	Ref.
Ru SAs-MoO_{3-x}/NF	209@10	55.0	3.5	*This work*
Au/Ni_3S_2/NF	230@10	51	4.97	*Appl. Catal B,2022, 304,120935*
Ir-NR/C	296@10	60.3	0.283	*Appl. Catal B,2020, 279,119394*
Ir/WOx/rGO	265@10	62	0.708	*Energy Environ Mater,2020, 4 (4): 681-686.*
NiIr-LDH	279@100	-	1.0	*J Am Chem Soc,2022, 144 (21): 9254-9263.*
Ni-Bi/meso-Ir	250@10	54.8	0.2	*Small Methods,2021, 5 (10): e2100679.*
Ru-NiFe-P/NF	227@50	66.1	-	*Appl. Catal B, 2020, 263,118324*
Ru–$NiSe_2$/NF	210@10	60.5	3.0	*Small,2022, 18 (6):*

				e2105305.
Ru_1/D-NiFe LDH/NF	189@10	31	2.0	*Nat Commun,2021, 12 (1): 4587.*
Ru-NiFe-P/NF	227@50	66.1	0.8	*Appl. Catal B, 2020, 263,118324*
PtIr/IrOx-50 NWs/C	266@10	44	0.0153	*Small,2022, 18 (20): e2201333.*

Table S4. Comparison of HER performance for Ru SAs-MoO_{3-x}/NF with other reported electrocatalysts in 1 M KOH.

Catalysts	η@j (mV @ mA cm^{-2})	Tafel slope (mV dec^{-1})	Loading of catalyst (mg/cm^2)	Ref.
Ru SAs-MoO_{3-x}/NF	36@10	41.3	3.5	This work
Ir-NR/C	42@10	35.2	0.283	*Appl. Catal B,2020,*

				279,119394
Ir/WOx/rGO	53@10	43	0.708	*Energy Environ Mater,2020, 4 (4): 681-686.*
Au/Ni_3S_2/NF	97@10	72	4.97	*Appl. Catal B,2022, 304,120935*
Pt_1/N-C	46@10	36.8	0.25	*Nat Commun,2020, 11,1029.*
Pd/NiFeOx/NF	76@10	78.03	-	*Adv. Funct Mater,2021, 31,51,2107181*
Ni_5P_4-Ru/CC	54@10	52	0.152	*Adv Mater,2020, 32 (11): e1906972.*
Ru1/D-NiFe LDH/NF	18@10	29	2.0	*Nat Commun,2021, 12 (1): 4587.*
Ru-NiFe-P/NF	44@10	80	0.8	*Appl. Catal B, 2020, 263,118324*
Pd, Ru–MoS_2−x(OH)y	48@10	45	0.357	*Nat Commun,2020, 11 (1): 1116.*
PtIr/IrOx-30 NWs/C	20@10	38	0.0153	*Small,2022, 18 (20): e2201333.*
Ni-Bi/meso-Ir	27@10	30.9	0.2	*Small Methods,2021, 5 (10): e2100679.*
Ru–$NiSe_2$/NF	59@10	72.2	3.0	*Small,2022, 18 (6): e2105305.*

Pt/MOF-O	66@10	101.6	0.0204	*J Am Chem Soc,2021, 143 (40): 16512-16518.*
Ru@Ni-MOF/NF	22@10	40	1.2	*Angew. Chem. Int. Ed. 2021, 60 (41): 22276-22282.*
Ru/P-TiO_2	27@10	28.3	0.416	*Angew. Chem. Int. Ed. 2022, 61, e202212196.*
Ru/TiO2-VO@C-15	64@10	58	0.571	*J. Mater. Chem. A, 2021, 9, 10160-10168.*

Table S5. Comparison of OWS performance for Ru SAs-MoO_{3-x}/NF with other reported electrocatalysts in 1 M KOH.

Catalysts	Voltage @ j (V @ mA cm^{-2})	Loading of catalyst (mg/cm^2)	Ref.
Ru SAs-MoO_{3-x}/NF || Ru SAs-MoO_{3-x}/NF	1.487@10<br>1.716@100	3.5	*This work*
Au/Ni_3S_2/NF	1.52@10	4.97	*Appl. Catal B,2022, 304,120935*
Ir-NR/C	1.57@10	0.283	*Appl. Catal B,2020,*

			279,119394
Ir/WOx/rGO	1.53@10	0.708	*Energy Environ Mater,2020, 4 (4): 681-686.*
Pd/NiFeOx/NF	1.57@20	-	*Adv. Funct Mater,2021, 31,51,2107181*
Ru1/D-NiFe LDH/NF	1.44@10	2.0	*Nat Commun,2021, 12 (1): 4587.*
Ru-NiFe-P/NF	1.47@10	0.8	*Appl. Catal B, 2020, 263,118324*
PtIr/IrOx-30 NWs/C‖PtIr/IrOx-50 NWs/C	1.52@10	0.0153	*Small,2022, 18 (20): e2201333.*
Ni-Bi/meso-Ir	1.55@10	0.2	*Small Methods,2021, 5 (10): e2100679.*
Ru–$NiSe_2$/NF	1.53@10	3.0	*Small,2022, 18 (6): e2105305.*

Table S6. Comparison of OWS performance for Ru SAs-MoO_{3-x}/NF with other reported electrocatalysts in alkaline seawater media.

Catalysts	Electrolytes	Voltage @ j (V @ mA cm^{-2})	*Ref.*
Ru	1 M KOH + natural	1.759@100	*This work*

SAs-MoO_{3-x}/NF ‖ Ru SAs-MoO_{3-x}/NF	seawater		
Ru MOF CoFe/CC	1 M KOH + natural seawater	1.54@10	*Nanoscale,2022, 14 (17): 6557-6569.*
1D-Cu@Co-CoO/Rh	1 M KOH + natural seawater	1.70@10	*Small,2021, 17 (50): e2103826.*
NiFe-PBA-gel-cal	1 M KOH + simulated seawater	1.66@100	*Adv Sci,2022, 9 (15): e2200146.*
Ni_{SA}-Ni_{Pi}/MoS_2 NSs(+,-)	1 M KOH + natural seawater	1.66@10	*Energy Environ Mater,2022 , 10.1002/eem2.12366*
Ir_1/$Ni_{1.6}Mn_{1.4}O_4$ ‖ Pt/C	1 M KOH + natural seawater	1.62@100	*Adv Sci,2022, 9 (16).*
NCMS/NiO	1 M KOH + natural seawater	1.757@100	*J. Mater. Chem. A,2022, 10 (17): 9547-9564.*
Ni3FeN@C/NF ‖ Ni3N@C/NF	1 M KOH + natural seawater	1.69@100	*J. Mater. Chem. A,2021, 9 (23): 13562-13569.*
S,P-(Ni,Mo,Fe)OOH/NiMoP	1 M KOH + natural seawater	1.741@100	*Appl. Catal B,2021, 293,120215*
Co-Fe2P	1 M KOH + simulated seawater	1.69@100	*Appl. Catal B,2021, 297,120386*
NiCoS ‖ NiMoS	1 M KOH + natural	1.73@100	*Appl. Catal B,2021,*

	seawater		*291,120071*
RuV–CoNiP/NF	1 M KOH + natural seawater	1.809@100	*J. Mater. Chem. A,2021, 9 (47): 26852-26860.*

Table S7. Comparison of bifunctional performance for Ru SAs-MoO_{3-x}/NF with other reported electrocatalysts in alkaline seawater media.

Catalysts	Loading of catalyst (mg/cm^2)	OER η@j (mV @ mA cm^{-2})	HER η@j (mV @ mA cm^{-2})
Ru SAs-MoO_{3-x}/NF	This work	230@10	43@10
RuV–CoNiP/NF	3.6	318@100	103@100
1D-Cu@Co-CoO/	-	260@10	137.7@10

Rh			
NiFe-PBA-gel-cal	0.057	329@100	480@100
Ni_{SA}-Ni_{Pi}/MoS_2 NSs(+,-)	7.17	314@10	94@10
Ni3FeN@C/NF	4.0	313@100	142@100
S,P-(Ni,Mo,Fe)OOH/NiMoP	1.0	279@100	143@100
Co-Fe2P	2.0	274@100	221@100
NiCoS	14.3	360@100	145@100
